# Personalized medicine in psychiatry: problems and promises

**DOI:** 10.1186/1741-7015-11-132

**Published:** 2013-05-16

**Authors:** Uzoezi Ozomaro, Claes Wahlestedt, Charles B Nemeroff

**Affiliations:** 1 University of Miami, Leonard M. Miller School of Medicine, Miami, FL, USA; 2 Center for Therapeutic Innovation, Hussman Institute for Human Genomics, University of Miami Miller School of Medicine, Miami, FL, USA; 3 Department of Psychiatry and Behavioral Sciences, University of Miami, Leonard M. Miller School of Medicine, Miami, FL, USA

**Keywords:** Major depressive disorder, Schizophrenia, Personalized medicine, Psychiatric hereditability, Epigenetics, Environmental factors, Endophenotypes, Pharmacogenomics, Neuroimaging genetics

## Abstract

The central theme of personalized medicine is the premise that an individual’s unique physiologic characteristics play a significant role in both disease vulnerability and in response to specific therapies. The major goals of personalized medicine are therefore to predict an individual’s susceptibility to developing an illness, achieve accurate diagnosis, and optimize the most efficient and favorable response to treatment. The goal of achieving personalized medicine in psychiatry is a laudable one, because its attainment should be associated with a marked reduction in morbidity and mortality. In this review, we summarize an illustrative selection of studies that are laying the foundation towards personalizing medicine in major depressive disorder, bipolar disorder, and schizophrenia. In addition, we present emerging applications that are likely to advance personalized medicine in psychiatry, with an emphasis on novel biomarkers and neuroimaging.

## Introduction

**Figure 1:**
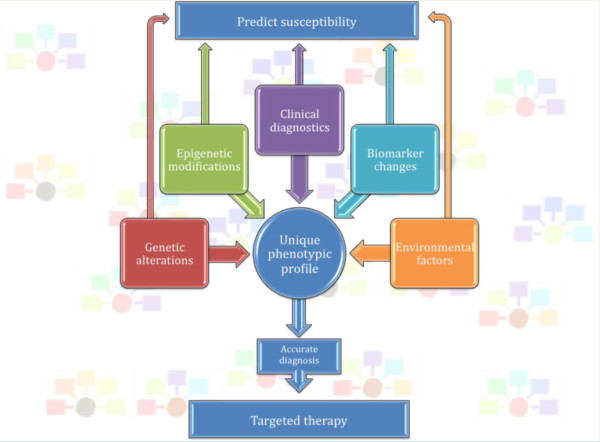
**Personalized medicine.** Forefront shows the schematic of the various factors that play into developing a unique phenotypic profile: genetic alterations, epigenetic modifications, clinical diagnostics, biomarker changes, and environmental changes. Upon obtaining a unique phenotypic profile, the psychiatrist is in a better position to either predict susceptibility to disease or make an accurate diagnosis. This, is in turn, allows for therapy targeted to the individual. Background: each individual will have differences in these components, giving rise to a unique phenotypic profile.

The foundation of personalized medicine centers on the assumption that an individual’s unique characteristics play a significant role in tailoring their therapies. Such characteristics include: genetic alterations and epigenetic modifications, clinical symptomatology, observable biomarker changes, and environmental factors [1]. The goals of personalized medicine are to predict the individual’s susceptibility to disease, achieve an accurate diagnosis, and result in an efficient and favorable response to treatment (Figure 1). Although there are clearly some very successful examples of personalized medicine, especially in oncology, relatively few such examples exist in psychiatry [2]. In this review, we summarize an illustrative selection of the advancements toward personalizing medicine in major depressive disorder (MDD), bipolar disorder (BD), and schizophrenia (SZ). We also discuss some new approaches currently being used and how they are likely to affect the field in the years to come.

## Contributing factors to psychiatric heritability

### Genetics

One major expectation of personalized medicine is the ability to determine susceptibility or protective factors imparted through genetic change. Interestingly, in the era of genome-wide association studies (GWAS), the majority of replicable findings do not pinpoint common genes underlying susceptibility or protection from disease; instead, our current understanding centers primarily on rare genetic variants, although a number of common variants have furthered understanding as well. However, both common and rare variants account for relatively small percentages of heritability, and far greater percentages are still attributed to ‘missing heritability’. Of the diseases reviewed in this report, no variation confers an autosomal dominant mendelian inheritance typical of Huntington’s disorder [3]; moreover, no single genetic change has shown an effect on heritability percentage that is in double digits. Nonetheless, there have been seminal genetic findings, each deepening our understanding of the major psychiatric illnesses. A sample of such findings in MDD, BD, and SZ is given below.

### Genetics: major depressive disorder

MDD has a strong genetic component, with an estimated 40 to 70% of the risk for developing MDD thought to be genetic [4]. Prominent findings in susceptibility studies of MDD include several polymorphisms in the serotonergic system, and in various elements of the hypothalamic-pituitary-adrenocortical (HPA) axis.

Genes involved in the serotonergic neural system have been intensively scrutinized in candidate gene and linkage studies in MDD for a number of reasons: this system is a major target of several antidepressants, and patients with MDD have alterations in multiple components of the serotonin system [5,6]. Polymorphisms and variable number tandem repeat regions (VNTR) in the 5-hydroxytryptamine (5HT; serotonin) transporter (*5-HTT*) gene have been associated with development of MDD. In 1996, Ogilvie and colleagues identified three novel alleles of a 5-HT VNTR region, and identified an association between the nine-copy VNTR allele and risk for developing MDD [7]. In a group of 466 German patients with MDD and 836 controls, Hoefgen *et al*. [8] reported a significant increased frequency of a 44-base pair insertion/deletion polymorphism in the 5′ promoter region of the *5-HTT* gene (5-HT transporter-linked polymorphic region; *5-HTTLPR*) in patients with MDD relative to controls. Furthermore, this *5-HTTLPR* polymorphism has been studied extensively both in the relationship between MDD and environmental factors and in the pharmacologic response to treatment (as discussed in the sections on ‘Environmental factors’ and ‘Prediction of treatment response,’ respectively).

A rate-limiting enzyme involved in serotonin synthesis, tryptophan hydrolylase (TPH), has been implicated in susceptibility for MDD by a number of reports, although attempts at replication have shown discordant findings [9]. Homologs 1 and 2 of the TPH gene (*TPH1* and *TPH2*, respectively), have both been associated with MDD susceptibility. Although both TPH1 and TPH2 are involved in serotonin synthesis, their distribution is markedly different. TPH1 is found primarily in the periphery, with effects on melatonin synthesis, hemostasis, and immune system function. Conversely, TPH2 is expressed in the CNS, with central effects on sleep, aggression, food intake, and mood [10]. Two groups [11,12] analyzed *TPH1* single-nucleotide polymorphism (SNP) and haplotype differences between participants with depression and control participants. In the former study, Nash *et al*. related quantitative phenotypes of depression in a community-based sample of 119 sibling groups to genetic alterations in TPH1, and identified a significant association between MDD susceptibility and a microsatellite downstream of *TPH1*[11]. In the latter study, Gizatullin *et al*. conducted a genetic screen of 228 patients with MDD and 253 healthy controls, and identified six haplotypes that were associated with MDD [12].

With respect to *TPH2*, Zill *et al*. [13] identified two SNPs (rs1386494 and rs1843809) associated with MDD in a sample of 300 Caucasian patients with MDD and 265 healthy controls. Subsequently, Zhang *et al*. searched for a novel loss-of-function mutation, G1463A, which they had identified and characterized in the previous year in sample of 87 patients with MDD and 219 controls [14,15]. The authors found that this mutation was significantly more common in patients with MDD than in controls, and suggested that defects in brain serotonin synthesis may be an important contributor to MDD susceptibility [15]. A subsequent study by Garriock *et al*. [16] failed to replicate these findings in a population similar in ethnicity and gender distribution to that of Zill *et al*. [13] Serretti *et al*. evaluated *TPH2* SNPs in MDD, BD, and SZ in Korean psychiatric inpatients and controls; their findings suggested that *TPH2* SNPs are not associated with MDD, BD, or SZ [17]. In a meta-analysis of *TPH2* genetic polymorphisms and MDD, Gao *et al*. examined 74 *TPH2* SNPs published through the end of October 2011, and using fixed-effects modeling, they found that two SNPs, rs4570625 and rs17110747, were associated with MDD susceptibility. The relationship between SNP rs4570625 and MDD was more robust, remaining significant using more conservative random-effects calculations [9]. Notably, this SNP was not one of the SNPs reported in the Zill *et al*. or Zhang *et al*. studies, although there have been later reports suggestive of a minor role for rs4570625 in MDD as well as in SZ, panic disorder, obsessive-compulsive disorder, and attention-deficit hyperactivity disorder [18-22].

For almost five decades, hyperactivity of the HPA axis has been reported, and this is suggested to be contributory to depressive symptomatology [23]. Accordingly, the components of the HPA axis provides numerous genes that might be associated with risk for MDD, including, but not limited to, a key component of the glucocorticoid receptor complex, FK506-binding protein 5 (*FKBP5*), corticotropin-releasing hormone receptor 1 (*CRHR1*) and corticotropin-releasing hormone-binding protein (*CRHBP*) [1]. In 2004, Binder and colleagues evaluated several genes that might be responsible for the HPA hyperactivity characteristic of depression. They identified SNPs in *FKBP5* associated with increased recurrence of MDD episodes and to a more rapid therapeutic response to antidepressant therapy (discussed in the section ‘Prediction of treatment response’) [24]. A 2009 study by Tatro *et al*. genotyped two SNPs in *FKBP5*, rs3800373 and rs1360780, in 60 frozen brain samples distributed over five clinical groups: MDD, MDD with psychosis, HIV-positive with MDD, and HIV-positive and HIV-negative controls. The rs3800373-CC genotype was found significantly more frequently in the MDD and MDD with psychosis groups than would be expected based on published allelic frequencies. The authors also reported that rs1360780 allele frequencies differed significantly from the expected allelic frequencies in the MDD and MDD with psychosis groups [25]. Using a sample of 155 European adolescents with MDD from the TORDIA (Treatment of SSRI (selective serotonin reuptake inhibitor)-Resistant Depression in Adolescents) trial, Brent *et al*. provided preliminary reports of two *FKBP5* genotypes (rs1360780-TT and rs3800373-GG) significantly associated with suicidal events [26].

*CRHR1* codes for a G-protein-coupled receptor involved in the regulation of the HPA axis by mediating the effects of corticotropin-releasing hormone (CRH) [27]. Raised levels of CRH in regional brain and cerebrospinal fluid (CSF) are a consistently replicated finding in patients with depression, and is also seen in suicide victims [28-31], rendering *CRHR1* an attractive candidate gene for MDD susceptibility. In 2006, Liu *et al*. identified three SNPs in *CRHR1*, which were significantly more common in a group of 206 Han Chinese patients with MDD compared with 195 controls matched for age, gender and ethnicity [32]. Subsequently, Papiol *et al*., compared *CRHR1* SNP frequencies in 159 Spanish outpatients with MDD and 96 healthy controls, and found an association between the *CRHR1* SNP rs110402 and early age of MDD onset. This SNP was also associated with increased risk for a seasonal pattern of illness [33]. Following these findings, Lekman *et al*. analyzed clinical data from 1,809 outpatients with MDD and a collection of 739 ‘Black’ and ‘non-Hispanic White’ ethnically matched controls enrolled in the STAR*D (Sequenced Treatment Alternatives to Relieve Depression) study. These authors found that the SNP rs1360780 was associated with MDD susceptibility in the non-Hispanic White sample, and the SNP rs4713916 was associated with disease remission to citalopram in the overall patient sample [34].

A consistent finding that clearly contributes to the HPA axis hyperactivity in MDD is hypersecretion of CRH. The CRH-binding protein (CRHBP) regulates the availability of CRH, both centrally and in the systemic circulation, which modulates HPA axis activity. SNPs in *CRHPB* were first associated with MDD in a case–control study conducted in a Swedish population [35]. Claes *et al*. examined 89 Swedish patients with recurrent MDD and 88 control samples matched for age, gender and ethnicity, and found two SNPs that were marginally associated with MDD. The authors reported one haplotype block comprised of the *CRHPB* SNPs s02-TT, s11-TT, and s14-T, whose presence significantly increased susceptibility to MDD [35]. In 2007, Van Den Eede *et al*. sought to replicate the *CRHBP* Swedish study findings in an extended Swedish sample, and in a larger and ethnically distinct sample (Belgian population). They analyzed 317 patients with MDD and 696 controls, but were unable to detect any statistically significant association (capable of withstanding correction for multiple testing) between the *CRHBP* SNPs in either the extended Swedish sample or Belgian sample [36].

### Genetics: bipolar disorder

Evidence from family, twin, and adoption studies show that BD is highly heritable, with genetic variables estimated to account for 60 to 85% of risk [37]. Efforts to discover the genetic sources for BD risk have led to innumerable linkage studies, some of which have identified promising susceptibility loci. However, these linkage studies have been fraught with inconsistent replication and indeterminate genetic causes of increased linkage signal [38,39]. Hence, there have been increasing efforts toward association studies. As with MDD, genes of the HPA axis have been probed for candidates increasing susceptibility to BD. Willour *et al*. genotyped *FKBP5* SNPs in a family sample of 317 BD pedigrees and 554 affected offspring. They found evidence for an association with BD for five SNPs (rs4713902, rs7757037, rs9296158, rs3800373, and rs9380525), with rs4713902 showing the most robust signal (*P*= 0.0001). Furthermore, Willour and colleagues identified four SNPs (rs1043805, rs3800373, rs9296158, and rs1360780) in covariate-based analyses that showed differential association with BD depending on the covariates of attempted suicide and/or the number of depressive episodes [40]. However, other studies were unable to replicate the findings of association of BD with *FKBP5*[41,42]. Subsequent GWAS in BD have contributed modest evidence of BD susceptibility attributable to SNPs in *FKBP5*[43,44].

Many candidate genes have been derived from the dopaminergic, serotonergic, and noradrenergic systems, based on the evidence for a role of these circuits in BD pathogenesis. In particular, the genes encoding 5-HTT, monoamine oxidase A (*MAOA*) and catechol-O-methyltransferase (*COMT*) have generated both positive and negative association findings with BD. However, there is no conclusive evidence for indisputable association of any of these genes with BD susceptibility [39]. Circadian rhythm disturbances are commonly seen in BD, which has led to multiple studies of association between circadian rhythm genes and BD [38,45,46]. The genes coding for aryl hydrocarbon receptor nuclear translocator-like BmaL1 (*ARNTL*) and circadian locomotor output cycles kaput (*CLOCK*) are two genes partially responsible for control of the internal circadian clock in the suprachiasmatic nucleus of the hypothalamus [47]. Both of these genes have been identified in association studies of BD. For example, Mansour *et al*. compared 234 Caucasian individuals with BD with 180 community-based controls in a BD association study. The authors genotyped 44 SNPs from eight circadian rhythm genes: *ARNTL*, *CLOCK*, *Period 1*, *2*, and *3* (*PER1*, *PER2*, *PER3*), cryptochrome 1 and 2 (*CRY1* and C*RY2*) and *TIMELESS*. They found modest associations with SNPs for *ARNTL* and *TIMELESS*, although they cautioned that additional studies are necessary to corroborate the findings [48]. Shi *et al*. conducted an association study of 10 circadian genes using the Sibling-Transmission Disequilibrium Test (sib-tdt) in an extended family collection (composed of 70 trios and 237 quads) in BD, and identified nominally significant association of three SNPs near or within the *CLOCK* gene; however, these associations did not survive correction for multiple testing [46].

GWAS in BD have identified four genes for further study that had SNPs of genome-wide statistical significance: calcium channel, voltage-dependent, L type alpha 1C subunit (*CACNA1C*), ankyrin 3 (*ANK3*), neurocan (*NCAN*) and odd Oz/ten-m homolog 4 (*ODZ4*) [49-51]. Although not significant at the genome-wide level, spectrin repeat containing, nuclear envelope 1 (*SYNE1*), has been recently associated with BD and with recurrent MDD [52]. Green *et al*. tested *SYNE1* SNP rs9371601 in 1,527 subjects with BD and compared them with 1,579 non-psychiatrically screened controls, finding evidence for the association of the SNP with BD (*P*= 0.0095) [52]. Furthermore, they identified a significant association between the SNP and recurrent MDD in a sample of 1,159 subjects with recurrent MDD compared with 2,592 controls (*P*= 0.032). *SYNE1* codes for Nesprin-1, a protein comprising part of the scaffolding that links the nucleoskeleton to the cytoskeleton (LINC) [53]. The association findings in *SYNE1* relating to BD and to MDD are likely to spark subsequent genetic and functional studies.

### Genetics: schizophrenia

With estimates of heritability of 50 to 80%, SZ is one of the most heritable of the disorders discussed in this paper [54,55]. Innumerable candidate gene studies and more than a handful of GWAS have contributed to the impression that SZ, despite its high heritability, is genetically complex, probably with a large polygenic component explaining a substantial amount of susceptibility [56]. In fact, the International Schizophrenia Consortium (ISC) tested a polygenic model for SZ by summarizing nominally significant associations in their GWAS data into quantitative scores. They then related these derived scores to disease states in three large, independent target samples [57]. From these analyses, the ISC concluded that one-third of genetic susceptibility for SZ lies in the collective effect of hundreds or thousands of common polygenic variants, each contributing small effects [56,57]. GWAS are poised to reveal such variants, and the results of the SZ GWAS are largely in agreement with the ISC assessment. Three comprehensive analyses of GWAS data from the ISC, Molecular Genetics of Schizophrenia (MGS) and the Schizophrenia Genetics Consortium (SGENE) have implicated a few discrete genes with odds ratios in the range of 0.73 to 1.23 [56-59]. These analyses also implicated the genome region (between 26 and 33 million bp) containing the major histocompatibility complex (MHC), a group of genes that encodes proteins necessary for the immune system to recognize foreign substances [56-59]. Clarifying the relationship between the MHC genes and SZ continues to be an area of active research.

GWAS and linkage studies have homed in on possible SZ-associated genes and genetic regions outside of the MHC region that have garnered interest, including the zinc finger 804A (*ZNF804A*) gene. The 2008 study of O’Donovan *et al*. GWAS identified 12 loci with ‘moderately strong’ (*P*<10^-5^) evidence for association in their primarily European-ancestry sample of 479 SZ and 2,937 controls. Subsequently, one SNP within the intron of the *ZNF804A* gene, rs1344706, remained significant in their larger replication samples (6,666 SZ subjects and 9,897 controls, including 1,782 Chinese and Japanese SZ subjects, and 1,865 controls) at the more stringent ‘strong’ threshold of *P*<5 × 10^-7^ (*P* = 1.61 × 10^-7^) although not at genome-wide significance threshold (*P*<7.2 × 10^-8^) [60]. The authors then broadened their phenotype to include BD, and found a strengthening of this association beyond the genome-wide significance threshold (*P* = 9.96 × 10^-9^) [60]. Several independent replication studies identified at least nominal associations between *ZNF804A* and SZ [61-63]. A 2010 case–control study by Riley *et al.*, compared 1,021 Irish subjects with SZ or poor-outcome schizoaffective disorder with 626 Irish control subjects, and reported nominally significant association between SZ and *ZNF804A* rs1344706 (*P* = 0.0227) and two other *ZNF804A* SNPs (rs17508595 at *P* = 0.0230 and rs7597593 at *P* = 0.0013) [61]. Steinberg and colleagues (2011) reported an association between rs1344706 and SZ (*P* = 0.0029) and SZ plus BD (*P* = 0.00065) in their sample (European ancestry with Chinese in follow-up study). Notably, when stratified by population, only two of the thirteen ethnicities, that is, Danish and Russian, exhibited a significant association between rs1344706 and SZ [62]. Zhang *et al*. conducted a population case–control study and a family-based study in a Han Chinese population. They reported a significant over-representation of the T allele of rs1344706 in their SZ population relative to controls in the population-based study (*P* = 0.00083) and a trend toward positive association in their trios (*P* = 0.058) [63]. In the wake of these findings, Williams *et al*. performed a meta-analysis of the data from the discovery GWAS along with data from thousands of additional European-ancestry samples from in-house sources, five GWAS (ISC, MGS, SGENE-plus, the Lencz *et al*. GWAS, one unpublished Swedish GWAS from Sullivan and Hultman [57-59,64,65]), and a handful of collaborative groups (totaling 18,309 SZ cases and 35,739 controls). The meta-analysis supported the original findings for association between rs1344706 and SZ (*P* = 2.5 × 10^-11^; odds ratio (OR) = 1.10; 95% confidence interval (CI) 1.07 to 1.14) and with SZ plus BD (*P* = 4.1 × 10^-13^; OR = 1.11; 95% CI 1.07 to 1.14) [65]. To date, ZNF804A remains one of the most promising of the GWAS findings in SZ.

Another gene with genome-wide association with SZ is transcription factor 4 (*TCF4*), which is a gene important for neurodevelopment [59,66,67]. Mutations in this gene have been linked to Pitt-Hopkins syndrome, a neurodevelopmental condition characterized by mental retardation, epilepsy, hyperventilation episodes, and distinct facial features [67,68]. With respect to SZ, one *TCF4* intronic SNP (rs9960767; C allele), was found to be significantly associated in a GWAS and analysis of nearly 50,000 subjects (data from SGENE-plus, follow-up samples, ISC and MGS GWAS samples) (*P* = 4.1 × 10^-9^; OR = 1.23; 95% CI 1.15 to 1.32) [59]. Steinberg *et al*. (2011) expanded on these findings, evaluating SNPs that had *P*-values of less than 1 × 10^-4^ in the combined sample (SGENE-plus, MGS, ISC dataset) of Stefansson *et al*. [69]. In the Steinberg *et al*. study, an additional 9,246 Caucasian SZ samples (SZ, schizoaffective disorder, or persistent delusional disorder) and 22,356 controls were assessed, followed by analysis of another 1,014 SZ and 1,144 control samples in a subsequent follow-up. These analyses implicated the C allele of the original rs9960767 SNP in SZ, in the primary, but not in the secondary follow-up (*P* = 2.5 × 10^-4^; OR = 1.16; 95% CI 1.07 to 1.25 and *P* = 0.5; OR = 1.09; 95% CI 0.85 to 1.41, respectively) [69]. Considered together, the Stefansson *et al.* combined sample and the two follow-up studies yielded a genome-wide significant association of rs9960767-C with SZ (*P* = 4.2 × 10^-9^; OR = 1.20; 95% CI 1.135 to 1.27) [69]. In 2011, Ripke *et al*. completed a replication study in Caucasians that also identified association of *TCF4* with SZ (*P =* 2.29 × 10^-5^), albeit with a different SNP, rs12966547 (which is not in linkage disequilibrium (LD) with rs9960767) [70]. Analysis of their replication data pooled with their discovery sample (which has significant overlap with the Stefansson *et al*. sample), yielded an association of genome-wide significance (*P =* 2.6 × 10^-10^) [70]. Notably, a *TCF4*-SZ association has also been established in a non-Caucasian population. Li *et al*. explored the *TCF4* gene in a Han Chinese population of 2,496 subjects with SZs and 5,184 controls [71]. Interestingly, the *TCF4* SNP highlighted by Stefansson *et al*. is polymorphic in Caucasians, but is not polymorphic in the Chinese population [59,71]. Instead of using this SNP, Li *et al.* substituted a nearby SNP, rs2958182 (which is in complete LD with rs9960767, and is polymorphic in the Chinese population). This group found rs2958182 to be significantly associated with SZ in their Chinese population (*P* = 3.64 × 10^-6^), which provides additional support for a *TCF4*-SZ association [71]. Together, these findings continue to fuel genetic and functional exploration of *TCF4* in SZ.

Neuregulin 1 (*NRG1*) is a gene that is also involved in many neurodevelopmental functions, including regulation of the expression and activation of glutamate receptors, neuronal migration, and oligodendrocyte development [72-76]. Mutant mice heterozygous for NRG1 (or for its receptor, ERBB4) have fewer NMDA receptors than their wild-type littermates. Moreover, they exhibit a SZ-like phenotype, which is reversible with the atypical antipsychotic drug, clozapine [77]. Linkage studies in the 1990s and early 2000s originally highlighted the cytogenic region containing the *NRG1* gene, 8p22-21, as a possible ‘schizophrenia susceptibility locus’ (SSL) [78-81]. A more specific gene-disease association was first suggested in a 2002 Icelandic linkage study in which a *NRG1* haplotype, HapICE (composed of five SNPs and two microsatellite markers), was found significantly more often in SZ samples than in control samples [77]. These initial findings spurred more than 60 additional studies of *NRG1* as a putative schizophrenia risk gene, yielding mixed findings [82]. By 2011, several large case–control studies [83-88] (>1000 subjects) had found at least one positive *NRG1*-SZ association, whereas comparably sized studies found no association at all [89-94]. *NRG1* analyses in large family-based studies (>200 families) fared similarly, with nearly equal positive [95-99] and negative [41,100-102] association findings. Meta-analyses by Munafò and colleagues in 2005 and updated in 2008 [103,104], noted a gene-level association between *NRG1* and SZ, but their data did not support an association between any particular *NRG1* SNP or haplotype with the disease. The results suggest that analyses at the level of the gene, rather than the level of an individual SNP, functional variant, or haplotype, might be a more reliable method of analysis [104].

Over 8,000 polymorphisms spaced over more than 1,000 candidate genes have been systematically analyzed in SZ (full results of the analyses can be found in the comprehensive SZ association study database, SZGene; http://www.szgene.org) [82]. Notably, Allen *et al.* highlighted SNPs in 16 genes with at least nominally significant effects (OR ranging from 0.7 to 1.52): *DRD2*, *GRIN2B*, *TPH1*, *DTNBP1*, *MTHFR*, *DRD1*, *APOE*, *DRD4*, *COMT*, *TP53*, *HP*, *DAO*, *SLC6A4*, *GABRB2*, *PLXNA2*, and *IL1B*. [82]. One of these genes receiving a great deal of attention is *COMT*, in part because of its known functions, and in part due to its location in the genome. The *COMT* gene encodes a protein responsible for the metabolic degradation of catecholamines. In addition, *COMT* is located on chromosome 22, within a region that when deleted causes velocardiofacial syndrome. Notably, nearly 25% of the individuals inheriting the large deletion at 22q11.21 go on to develop SZ, making the inheritance of this deletion one of the greatest risk factors for development of SZ [105]. In 2002, Shifman *et al*. found a significant association between a *COMT* haplotype and SZ in a case–control study in Ashkenazi Jews [106]. One 5′-UTR SNP involved in the haplotype (rs737865) has been implicated in a subsequent studies, and despite both positive and negative findings, maintains nominal significance by SZGene meta-analysis, with a modest OR of 1.06 (95% CI 1.01 to 1.11, *P*<0.05) [82].

The disrupted-in-schizophrenia (*DISC*) locus has also been associated with SZ after its initial characterization in a large Scottish family with high psychiatric load [107]. The *DISC* locus was identified at the breakpoint of a balanced translocation between chromosomes 1 and 11. The translocation significantly segregated with a range of psychiatric illness in the family, including SZ, MDD, and BD [107]. Further analysis showed that the translocation is in significant LD with SZ, outside of the effects of the other psychiatric illnesses (log of the odds ratio score being 3.6) [108]. Fine mapping focused further studies on two genes, *DISC1* and *DISC2*, identified along the chromosome 1 component of the translocation [109]. *DISC1* codes for a protein known to have diverse roles, including regulation of neuronal axonal and dendritic outgrowth, cell proliferation, and cell differentiation [110]. *DISC2* is a non-protein-coding gene thought to be important in the regulation of *DISC1*[108,111]. The majority of *DISC1* family and association studies have had positive findings of a relationship between the gene and SZ [112-130]. However, there have also been a number of studies which have not been able to show any significant findings [41,93,131]. To date, there have not been any positive association studies for *DISC2* and SZ despite one large association study of 1,054 Scottish individuals (328 with SZ, 726 controls), which did not find any significant associations between *DISC2* SNPs and SZ [132].

### Epigenetic modifications

**Figure 2:**
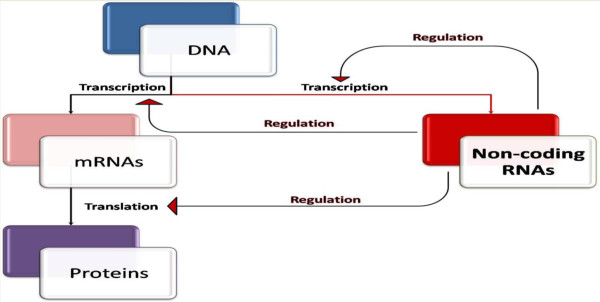
**‘Modified’ central dogma.** The central dogma of molecular biology states that DNA is transcribed to RNA, which is then translated into protein. In light of the emerging importance of non-coding RNAs, this diagram shows how non-coding RNAs serve to regulate each step in the central dogma, including regulating their own transcription.

Although genetics clearly play an important role in personalized medicine, the role of epigenetics should not be overlooked. Epigenetics refers to the phenomenon of heritable changes to gene regulation caused by mechanisms that do not involve modification of the nucleotide sequence. Increasingly, it is being realized that these mechanisms are far more complex than originally envisioned, requiring adjustment of the current perspective on the central dogma of molecular biology; namely, that DNA transcribes RNA, which is subsequently translated into protein [133]. It is estimated that approximately 98% of the human genome is not translated into protein, and these epigenetic changes occur within the framework of DNA being transcribed into both coding and non-coding RNA, with the subsequent translation of coding RNA into protein, and the concurrent regulation of each of these steps by non-coding RNA [134,135] (Figure 2). Specific epigenetic mechanisms include DNA methylation, histone modification, alternative splicing, RNA editing, and non-transcriptional gene silencing via microRNAs [136]. Epigenetic regulation has been implicated in a myriad of neural processes, from learning and memory to seizures and neurogenesis, and recently to depression, chronic stress, and various addictions [137,138].

### Epigenetic modifications: major depressive disorder

Study of the epigenetics of MDD, particularly involving DNA methylation and histone modification, has generated some interesting findings [6]. Electroconvulsive therapy (ECT), the induction of controlled seizures to relieve depressive symptoms, can be remarkably effective in severe treatment refractory cases of depression [139]. The effectiveness of ECT requires multiple treatments, and without continued antidepressant therapy or maintenance, nearly all patients will have a relapse of their symptoms [138,139]. Epigenetic changes are thought to partially account for this phenomenon.

In animal models of depression, chromatin remodeling of brain-derived neurotrophic factor (*BDNF*) promoters leads to site-specific increased *BDNF* transcription, a change shown to mediate susceptibility to stress in humans and in mouse models [6,140]. Another notable example of epigenetic changes is seen in rats reared by mothers who exhibit especially high levels of nurturing behaviors of pup licking and grooming (LG) [141]. The authors described the adult rat offspring from high-LG mothers as being less anxious than their low-LG counterparts. The high-LG offspring also displayed corresponding measurable changes to the HPA axis (for example, increased glucocorticoid receptor mRNA and protein expression, decreased hypothalamic corticotropin-releasing factor (*CRF*) mRNA expression and increased sensitivity to glucocorticoid negative feedback), changes that are due, in part, to gene-expression changes that remain stable until adulthood. Of note, these gene-expression alterations are reversible with cross-fostering of rats between high-LG and low-LG mothers [138,141-143]. Building on these principal findings, McGowan *et al*. [144] explored the epigenetic regulation of a neuron-specific glucocorticoid receptor gene (nuclear receptor subfamily 3, group C, member 1; *NR3C1*) in human suicide victims subjected to childhood abuse. In their study, they compared post-mortem hippocampal tissue samples from 12 suicide victims with a history of childhood abuse, 12 suicide victims with no history of childhood abuse, and 12 controls (persons experiencing sudden, accidental death without a history of childhood abuse). They found that the expression of total glucocorticoid receptor mRNA was significantly lower in the abused suicide victims than in either the non-abused suicide victims or the controls. Furthermore, the level of methylation of the *NR3C1* promoter sites was significantly higher in the abused suicide victims compared with the non-abused suicide victims or controls. Notably, in both of these findings, there were no significant differences between the non-abused suicide victims and the controls. The authors report that these findings are consistent with those in animal models showing epigenetic modifications of the glucocorticoid receptor leading to increased HPA responses to stress. Moreover, they speculate that early adverse life events, such as childhood abuse, may lead to epigenetic changes that contribute to risk for developing psychopathology [144].

### Epigenetic modifications: bipolar disorder and schizophrenia

Because many epigenetic studies of BD also include patients or tissue samples with SZ, the epigenetic studies involving both disorders are summarized here. The role of methylation in SZ has been suggested for over 50 years, when patients with SZ who were administered a methylating agent, methionine, were noted to experience exacerbation of their psychosis [145,146]. Since that time, many post-mortem brain studies have focused on methylation patterns in SZ. For example, differential methylation patterns in the regulatory regions of *COMT* and reelin (*RELN*) have been found between individuals with SZ compared with controls. From a functional standpoint, dysregulation of either of these genes could feasibly result in psychopathology. *COMT* is an important gene for metabolizing catecholamines, while *RELN* encodes an extracellular matrix glycoprotein theorized to control cell-cell interactions during neuronal positioning and migration during brain development [147]. Abdolmaleky and colleagues analyzed 115 post-mortem brain samples from the frontal lobe of individuals with BD or SZ and controls (35 samples per group). The membrane-bound (*MB*)-*COMT* promoter was hypomethylated significantly more often in the brains of individuals with SZ or BD relative to the brains of controls [148]. Generally, methylating DNA sequences tends to decrease their transcription, and accordingly, this hypomethylation in the BD and schizophrenic brains corresponded with higher *MB-COMT* transcript expression than seen in the controls [148]. The authors remarked that the resulting overexpression of *MB-COMT* could lead to significant increases in dopamine degradation. Moreover, the resulting hypodopaminergic state and frontal-lobe hypoactivity may be amenable to COMT-inhibitor drugs or preventative strategies to modulate methylation of the *COMT* promoter [148]. Lower levels of *RELN* mRNA expression and protein concentrations were found in post-mortem brain samples from patients with schizophrenia relative to control brain samples, which led to investigations of whether promoter regions are differentially methylated in such patients relative to controls [149]. In a small (*n* = 10) post-mortem study of schizophrenic brains compared with controls, Abdolmaleky *et al*. found a significant increase in DNA methylation of CpG islands in the *RELN* promoter in brains from individuals with SZ compared with controls [150]. However, neither set of findings (the hypomethylation of the *COMT* regulatory regions and the hypermethylation of the *RELN* regulatory regions) have been replicated in subsequent studies [151,152].

Some studies have suggested that epigenetic dysregulation of the 5-HT2A receptor gene (*HTR2A*) may contribute to the pathophysiology in SZ. The focus on *HTR2A* is not surprising, as it has been the subject of many studies, which have provided conflicting evidence for a small, but significant, association of an *HTR2A* SNP, rs6313 (c.102C>T), with SZ in European populations [82,153-155]. This 102-nucleotide position is near the promoter region, and is thought to be a putative methylation site, which is consistent with the lower levels of *5HT2A* mRNA seen with the C allele (in both patients and controls). Indeed, in a study on post-mortem human brain tissue, Polesskaya *et al.* reported that one of the key determinants of *HTR2A* expression was cytosine methylation at rs6313 [156]. Notably, this decrease in *HTR2A* mRNA is a property that is exaggerated in SZ [153,154]. Furthermore, antipsychotic treatment decreases methylation at the 102C polymorphic site, while *HTR2A* mRNA expression increases [157]. Interestingly, this SNP is in complete LD with a promoter SNP, rs6311 (c.-1438G>A), for which there is also some evidence in favor of an association with SZ in Caucasian samples (SZGene 2010 meta-analysis: OR = 1.20; 95% CI 1.07 to1.36]) [82,158]. Carrying the minor allele in rs6311 results in the creation of a methylation binding site for the E47 transcription activator at position −1,438, and results in the loss of a CpG binding site at position −1,439 [158]. These findings indicate how genetic alteration can trigger a chain of downstream epigenetic effects. Clearly, deciphering the effects of *HTR2A* gene-expression dysregulation in SZ will continue to be a rich source of study.

Histone modification is another epigenetic mechanism hypothesized to contribute to the pathogenesis of SZ and BD. Post-mortem studies have shown increased levels of histone deacetylase, type 1 (HDAC1), an enzyme generally responsible for silencing gene expression, in the pre-frontal cortices of patients with SZ [159-161]. Sharma *et al.* found a strong negative correlation between *HDAC1* and *GAD67* mRNA, which encodes an isoform of glutamate decarboxyase (GAD) [160]. Taken together, these findings provide a possible mechanism for the common observation of decreased expression of GAD, the rate-limiting enzyme involved in the synthesis of gamma-aminobutyric acid (GABA) in the brains of patients with schizophrenia or BD [162-165]. Furthermore, in an animal model that used methionine to induce SZ-like behavioral abnormalities, HDAC inhibitors (HDACi) were sufficient to attenuate the behavioral abnormalities [166]. Valproate is a mood stabilizer and a potent HDACi, whose mood-stabilizing effects in BD and SZ may be based on its epigenetic properties [167].

MicroRNAs (miRNAs) are important contributors to epigenetic modifications. Specifically, miRNAs are non-coding RNA sequences that can broadly regulate gene expression post-transcription [168]. Studies of miRNA in BD and SZ have thus far yielded somewhat inconsistent results [169]. Perkins *et al*. observed the expression pattern of miRNAs in the pre-frontal cortex (PFC), and identified 16 miRNAs differentially regulated in their sample of 15 patients with SZ or schizoaffective disease, relative to their 21 control samples. Of the 16 identified miRNAs, 15 were downregulated in SZ [170]. Studies conducted by Beveridge and colleagues in 2008 and 2010 showed miRNA dysregulation and altered miRNA biogenesis in the brains (specifically, the PFC and the subgenual superior temporal gyrus) of patients with SZ [171,172]. Interestingly, many of their identified miRNAs were upregulated, and those that overlapped with the Perkins study were upregulated in the Beveridge studies but downregulated in the Perkins study [170-172]. Furthermore, the identified alterations in miRNA in the Perkins study suggested a decrease in global miRNA biosynthesis whereas the 2010 Beveridge study suggested a global increase in miRNA biosynthesis in SZ [170,172]. A recent study by Miller *et al*. explored the expression of over 800 miRNAs in the dorsolateral PFC (dlPFC) in RNA samples from 35 patients with SZ, 31 with BD, and 34 controls [173]. The authors identified significant dysregulation in 10 miRNAs in BD and alteration of the miRNA-132 in SZ. Notably, there were no significant miRNA overlaps between BD and SZ. Further analyses, which ranked the associations of miRNA with BD or SZ by fold change and uncorrected *P*-values, identified six dysregulated miRNAs common to both conditions. In fact, 754 protein-coding genes are affected by two or more of these miRNAs, which is significantly higher than would be expected by chance (*P*<0.0001), suggesting that co-dysregulation of these targets may result in pathologic alterations in functionally overlapping molecular networks [173]. Reliably associating miRNAs with psychiatric illness is a relatively new field of study, although there are data to suggest that other individual miRNAs may be linked with SZ and or BD (for an excellent review, see Miller and Wahlestedt [169]).

### Clinical diagnostics: endophenotypes/biological markers

Endophenotypes are markers of an illness regardless of the phenotypic presence or absence of the illness. Because psychiatric disorders are primarily defined based on symptoms, many of which overlap between different disorders, the concept of defining endophenotypes for the disorders is an attractive one [174]. Gottesman and Gould in 2003 discussed endophenotypes in psychiatry and outlined criteria for defining endophenotypes. The endophenotype should segregate with the illness in the population, and co-segregate with the illness within families; it should be heritable and more prevalent in affected than in unaffected families; it should not depend on whether the illness is currently clinically manifested, and finally, it should be specific to the illness and capable of being reliably measured [174,175].

Biomarkers are measureable characteristics that reflect biologic function or dysfunction, response to a therapeutic measure, or indication of the natural progression of disease [176]. Historically, psychiatric illnesses have been diagnosed on the basis of behavioral signs and symptoms, rather than on biomarkers, but the overwhelming evidence that severe psychiatric disorders are in fact brain diseases, has led to considerable efforts to identify and introduce biomarkers into clinical psychiatry [177]. Current biomarkers for psychiatric illness fall into roughly three categories: metabolite levels, molecular genetics, and imaging findings. There are subtle distinctions between endophenotypes and biomarker changes, because endophenotypes are trait markers, whereas biomarkers may be state or trait markers [175]. However, there exists substantial overlap between these two categories, and we have therefore combined discussion of each into one section.

### Clinical diagnostics: major depressive disorder

Negative mood is a central feature in MDD, and there have been a number of studies evaluating this as an endophenotype for depression. Wichers *et al*. conducted a study in female twin pairs, assessing the relationship between daily life negative mood bias and the lifetime diagnosis of MDD [178]. They studied 259 twin pairs; 17.4% of the sample represented twin pairs of which one twin had a diagnosis of lifetime depression while the co-twin did not. The authors found that probands with co-twins meeting a diagnosis for lifetime depression exhibited greater negative affect responsiveness to daily life stressors. This relationship held true after controlling for proband continuous depression score, past depressive score, and exclusion of probands who were depressed at the time of the study. The authors concluded that increased negative affect reactivity is present in probands with high familial load for depression, regardless of past or current depression [178]. Furthermore, mood-congruent memory (MCM) bias was first reported in the 1980s and has been repeatedly demonstrated in MDD and dysphoria in a multitude of recent studies [179-183].

Similar to negative mood, anhedonia is a central feature of the MDD diagnosis and has been proposed as an endophenotype for MDD. Dryman and Eaton analyzed data from 49 individuals enrolled in the US National Institute of Mental Health Catchment Area Program (NIMH-ECA) during the period 1981 to 1985, and found that anhedonia often precedes the onset of MDD [184]. Furthermore, some researchers have found an association between family history for depression and anhedonia in unaffected relatives [185-187]. McCabe *et al*. used functional magnetic resonance imaging (fMRI) to compare 13 unmedicated individuals recovered from MDD with 14 healthy controls to evaluate whether deficits in the processing of reward-relevant stimuli are present in people at risk for developing MDD [188]. Compared with the healthy controls, the recovered MDD individuals showed decreased neural response in the ventral striatum to pleasant stimuli and an increased response in the caudate nucleus to aversive stimuli. The authors suggested that patients in remission from MDD may have deficits in the neural basis of reward [188].

CSF concentrations of the norepinephrine metabolite 3-methoxy-4-hydroxphenylglycol (MHPG) has long been proposed as a biomarker for suicide risk, although studies have generated conflicting findings [189,190]. In a 2009 study, Galfalvi *et al*. completed a prospective study of the relationship between MHPG levels and suicidal behavior in 184 subjects with MDD or BD experiencing a depressive episode. They identified that lower levels of the metabolite were predictive of suicidal behavior in this population. Additionally, lower MHPG levels correlated with higher medical lethality of the future suicide attempt. The authors suggested that CSF MHPG may be useful as a biomarker for risk for short-term suicidal behavior [190].

Neuroimaging findings are being increasingly proposed as endophenotypes in MDD. Ramel *et al.* used fMRI to compare 14 unmedicated remitted-depressed (RD) individuals with 14 matched, never-depressed (ND) individuals on a self-referent encoding/evaluation task completed before and after a sad mood challenge [191]. For a subset of the RD group, after priming with sad mood induction, bilateral amygdala activation predicted negative recall bias. Notably, this association was not present before the sad mood priming, nor was it present in the ND group for any mood state [191].

### Clinical diagnostics: bipolar disorder

BDNF is a protein that contributes to synaptic plasticity and neuronal survival, and has been implicated in the pathophysiology of a number of psychiatric illnesses [192,193]. Because BDNF levels in serum reportedly have high correlation (r = 0.8) with CSF levels, and are purported to cross the blood–brain barrier, BDNF is an attractive candidate for real-time study of BD mood states [194,195]. Recently, two meta-analyses by Fernandes *et al.*[196] and Lin [197] probed whether serum or plasma BDNF levels could reliably serve as a state marker of mood episodes in BD. In an analysis that combined studies of BDNF levels in either serum or plasma, Lin [197] identified that relative to controls, patients with BD in either manic or depressive states exhibited significant decreases in peripheral blood BDNF levels, and additionally, that pharmacologic treatment of manic states was accompanied by a significant increase in BDNF blood levels (plasma or serum). The Fernandes *et al*. [196] meta-analysis comprised 13 case–control studies (548 patients with BD and 565 controls), and measured serum BDNF levels during each of the three BD states: mania, euthymia, and depression. The analysis showed that, relative to levels in controls, serum BDNF levels were reliably decreased during manic and depressive states, and normal in euthymia. The Fernandes group suggested that serum BDNF concentrations can be used to accurately discriminate between the depressive episodes of MDD and those of BD (the suggested cut-off of 0.26 resulted in 88% sensitivity and 90% specificity) [198]. This has significant clinical implications because the ability to distinguish between BD and MDD is extremely important, especially in view of the likelihood of antidepressant monotherapy precipitating manic episodes in patients with BD [199].

Various cognitive phenotypes have also been suggested as endophenotypes for BD, including processing speed, visual learning and memory, verbal fluency, psychomotor retardation, and response inhibition [200]. In their meta-analysis of BD endophenotype candidates, Bora *et al*. [200] noted that of the 18 cognitive tests reviewed, euthymic patients with BD performed significantly more poorly than controls on all but one measure, the visual copy task. Moreover, the authors found that response inhibition is the most significant endophenotype for BD, which is significantly impaired in both patients with BD and unaffected relatives of such patients, but not in controls. Other proposed endophenotypes with strong support from their meta-analysis are verbal memory (large effect size in BD versus controls; modest effect size between relatives of patients with BD versus controls) and psychomotor retardation (moderate effect size in BD versus controls, no significant difference in relatives of patients with BD versus controls). The investigators recommended longitudinal studies to determine the relationship between cognitive impairment and brain connectivity and genetics [200].

### Clinical diagnostics: schizophrenia

The possibility that certain cognitive deficits should be considered as SZ endophenotypes has been supported by multiple lines of evidence [201,202]. Three meta-analyses reflect efforts to interpret which cognitive domains are most consistently deficient in the SZ population relative to controls [201,203,204]. The meta-analysis by Sitskoorn and colleagues [203] compiled data from 37 studies, and identified nine weighted variables (broadly derived from the three domains of attention, memory, and executive function) that differed significantly in SZ subjects relative to controls. The largest effect sizes found were for deficiency in verbal memory recall and executive functioning in subjects with SZ relative to healthy controls (Cohen’s d = 0.54, 95% CI 0.43 to 0.66, *P*<0.0001; d = 0.51, 95% CI 0.06 to 0.50, *P*<0.0001). Smaller effect sizes for their other variables ranged from d = 0.28 to d = 0.38 for the remainder of the variables (in decreasing effect size): visuomotor tracking, attention/verbal memory span, verbal fluency, sustained attention, visual memory, executive function, and selective attention [203]. Szöke *et al. *[204] reported in their meta-analysis six altered cognitive variables in first-degree relatives of subjects with SZ relative to controls, with the greatest effect sizes seen for semantic and phonological verbal fluency (Hedges’ unbiased g = 0.87, 95% CI 0.64 to 1.10; g = 0.65, 95% CI 0.48 to 0.82, respectively). The remainder of the effect sizes were smaller, ranging from g = 0.26 to g = 0.49 for visuomotor speed/executive function, selective attention, and executive function (Wisconsin Card Sorting Test categories and perseverative errors) [204]. The meta-analysis by Fioravanti *et al*. [205] identified five cognitive domains in which subjects with SZ performed significantly worse than controls: memory, language, attention, executive function, and IQ (with memory having the greatest standardized mean difference of −1.18 (95% CI −1.31 to−1.05, *P<*0.00001) [205]. In 2007, Gur *et al*. [202] assessed neurocognitive function in 349 members of 35 multigenerational families with SZ and in 154 medically and psychiatrically healthy controls in an attempt to characterize a neurocognitive profile for SZ. They found that probands with SZ were the most impaired with regard to certain neurocognitive domains, the control group was the least impaired, and the unaffected relatives of the probands with SZ were impaired at an intermediate level between the other two groups. In particular, the authors reported strong genetic influences on the variability of performance on memory and emotion processing accuracy and on speed of attention. Further, because these measures were associated with SZ, they could be applied to differentiate between unaffected relatives and controls, and because they appear to be heritable, the authors proposed that they could be suitable for use as endophenotypes for genetic studies [202]. Despite some heterogeneity between studies, others have identified cognitive deficits in individuals with SZ and their unaffected relatives compared with controls, consistent with generalized cognitive impairment in these patients and their genetically susceptible relatives [206-210].

### Environmental factors: stress

All of the reviewed psychiatric illnesses are characterized by various degrees of symptom exacerbation upon exposure to stress. Often underlying this response is dysregulation of the HPA axis, an important modulator of adaptive responses to stress. In fact, increased secretion of cortisol, one of the downstream products of the HPA axis, has been reported for decades in MDD [211,212]. Upstream of cortisol in the HPA axis is corticotropin releasing hormone (CRH, also known as corticotropin releasing factor, CRF), which is both an important regulator of the HPA axis and a key regulator in many extra-hypothalamic brain areas, including the amygdala, septum, stria terminalis, and cerebral cortex [213]. In these areas of the brain, CRH acts as a neurotransmitter, orchestrating endocrine, immune, and behavioral responses to stress [214]. In fact, injection of CRH into the CNS of rodents produces a host of endocrine and immune changes, and results in behavioral symptoms resembling those of MDD in humans [215,216]. In view of this, special attention has been given to identifying associations in HPA axis genes, in particular those affecting CRH levels. There have been numerous studies identifying SNPs in the gene coding for the CRH receptor 1 (*CRHR1*), which are associated with MDD [28]. Some studies have also identified differential treatment response to antidepressant therapy with various CRHR1 haplotypes. Importantly, certain *CRHR1* SNPs and haplotypes appear to modify the adult risk of MDD in individuals subjected to childhood trauma [217-219]. Bradley *et al*. [217] conducted a study in a group of primarily socioeconomically disadvantaged African-Americans with high rates of lifetime trauma (n = 422), to explore whether the effects of child abuse on MDD susceptibility is moderated by variation in the *CRHR1* gene. Linear regression analyses identified a significant protective effect of the gene and environment (G × E) interaction between two SNP haplotypes each with three SNPs (*P*<0.001; rs7209436-T, rs4792887-C, rs110402-A and *P*<0.005; rs7209436-T, rs242924-C and rs110402-A), and with the three individual SNP components of the latter haplotype. In an independent sample of primarily Caucasian individuals [217] (n = 299), the authors found similar findings, namely significant over-representation of the protective SNP alleles and haplotypes. Polancyzk *et al*. [218] replicated the Bradley findings in a sample of English women, but could not replicate the sample when using a different measure of child abuse than the Childhood Trauma Questionnaire (CTQ) used in the Bradley study. The *CRHR1* gene interaction with trauma in childhood and with MDD in adulthood has been scrutinized by two other groups, who used a dexamethasone/corticotropin-releasing hormone test (DEX/CRH) as a measure of HPA axis activity [219,220]. Tyrka *et al*. [219] found that two of the *CRHR1* SNPs identified in the Bradley *et al*. study [217], rs110402 and rs242924, showed a significant interaction with childhood abuse and prediction of cortisol response to the DEX/CRH challenge. Similarly, Heim *et al*. [220] used the DEX/CRH measure, and found the same relationship, although only in men, and suggested that the nature of the type of abuse experienced is crucial because in their sample, men experienced more physical abuse than did women.

In addition to its contribution to susceptibility for MDD and to epigenetic alterations in MDD (see ‘Genetic and epigenetics of MDD’) the serotonin transporter gene *5-HTT* polymorphism, *5-HTTLPR*, has been implicated in G × E interactions. Specifically, Caspi *et al*. [221] evaluated the relationship between MDD, the *5-HTTLPR* genotype, and stressful life events over five years in a group of 847 Caucasian non-Maori individuals involved in the Dunedin Multidisciplinary Health and Development Study prospective longitudinal study. The authors used a moderated regression framework with sex as a covariate, and reported that the effect of stressful life events was significantly stronger in individuals carrying the short allele than in those carrying the long allele (*P* = 0.02). Moreover, they identified that childhood maltreatment predicted adult depression in individuals carrying the short allele but not in individuals homozygous for the long allele (*P* = 0.05) [221].

### Environmental factors: in utero exposure

Much of the work focusing on *in utero* exposure to psychiatric illness has centered on SZ, with limited study of BD. Monozygotic twins are concordant for SZ in approximately 45% of cases, which indicates a potentially large contribution from environmental factors in the development of the illness [222]. Some studies have sought data from *in utero* and perinatal environmental exposures for potential explanations as to how monozygotic twins can be discordant for SZ. In 1967, the NIMH completed its first study on monozygotic twins discordant for SZ, examining 14 pairs of monozygotic twins, and summarizing published reports from 100 pairs of monozygotic twins discordant for SZ [223]. Comparing available information on the twin pairs illustrated that the SZ-affected twin was more likely to have sustained birth complications (index twin: control twin (I:C) = 4.0, n = 30), including perinatal asphyxia (I:C = 4.0; n = 15), and to be more than twice as likely to be the lighter-weight twin (I:C = 2.1; n = 61; *P*<0.01) [223]. These data laid the groundwork for many additional examinations of prenatal and perinatal insults. Cannon *et al*. [224] conducted a meta-analysis of eight prospective population-based studies of obstetric complications and SZ, and identified three groups of complications that significantly increased the risk for SZ: 1) pregnancy complications, including bleeding, Rhesus factor incompatibility, diabetes, and pre-eclampsia, 2) abnormal fetal growth and development, including low birth weight, congenital malformations, and reduced head circumference, and 3) delivery complications, including uterine atony, asphyxia, and emergency Cesarean section. Although many of the independent characteristics had modest effect sizes, with OR≥2 (bleeding, Rhesus factor incompatibility, pre-eclampsia, reduced head circumference, and asphyxia), other characteristics showed sizeable ORs: diabetes in pregnancy (OR = 7.76, 95% CI 1.37 to 43.90, *P*<0.03), low birth weight (< 2000g; OR = 3.89, 95% CI 1.40 to 10.84, *P* = 0.009), emergency Cesarean section (OR = 3.24, 95% CI 1.40 to 7.50, *P* = 0.006), congenital malformations (OR = 2.35, 95% CI 1.21 to 4.57, *P*<0.02), and uterine atony (OR = 2.29, 95% CI 1.51 to 3.50, *P*<0.001) [224]. By contrast, another study found no robust evidence for an association between BD and obstetric complications [225].

Other *in utero* exposures theorized to be involved in SZ are maternal influenza and maternal stress. Following an influenza pandemic in 1957, studies in Finland [226], England and Wales [227], and the USA [228] suggested a possible association between SZ development and prenatal exposure to the influenza virus. However, subsequent studies produced conflicting findings. A 2010 meta-analysis of 13 studies of SZ and maternal influenza exposure following the 1957 pandemic could not identify an association risk for SZ and prenatal influenza exposure [229]. The relationship between prenatal exposure to maternal stress and SZ was also analyzed by meta-analysis after individual studies produced equivocal findings. The meta-analysis by Selten *et al*. [230] could not identify a statistically significant association, despite querying various forms of maternal stress (wars, spousal demise, natural disasters, and undesired pregnancies).

## Prediction of treatment response or non-response (therapeutic and side-effect profile)

### Role of genetic alteration in drug-metabolizing enzymes

Many of the SNPs reviewed above and many others have been associated with predicting treatment response (both in terms of therapeutic efficacy and side-effect profile) to a pharmacologic intervention. Beyond the effects specific to particular illnesses or drugs are the genetic changes in drug-metabolizing genes that underlie differential response to pharmacologic agents. These genetic changes will be reviewed first, followed by specific polymorphisms in non-drug-metabolizing enzymes (non-DME) and their relationship to specific psychiatric illnesses.

The most studied group of drug-metabolizing enzymes (DME) in psychiatry are the cytochrome P450 (CYP) enzyme family [231]. CYP enzymes are expressed predominantly in the liver, although they are also found in many extra-hepatic locations, including in the brain, where their levels are approximately 0.5 to 2% of those in the liver [231]. Moreover, the genes coding for the CYP enzyme family are highly polymorphic, and the effects of many of the genetic differences contribute to differential metabolism of psychotropic agents [1,231]. DME phenotypes are broadly grouped into four categories based on genotype effect on enzyme activity: 1) poor metabolizers (PM), 2) intermediate metabolizers (IM), 3) extensive metabolizers (EM), and 4) ultra-rapid metabolizers (UM) [232]. CYP2D6 is an important member of the CYP enzyme family, because it is responsible for metabolizing nearly 50% of the most commonly prescribed psychotropics, and evidence in animal models indicates its involvement in the biosynthesis of dopamine and 5-HT [232-234]. It is also highly polymorphic, with 7% of Caucasians falling into the CYP2D6-PM group [231]. Common substrates metabolized by CYPD26 are tricyclic antidepressants, many SSRIs, venlafaxine, and antipsychotics. Therefore, when presented with any of these drugs, CYP2D6-PM are generally at risk for side effects secondary to the increased bioavailability and prolonged elimination half-lives of these drugs. Theoretically, these patients are also subject to an exaggerated drug response by the same mechanisms. At the other phenotypic extreme, the CYP2D6-UM would be expected to exhibit a poorer therapeutic response to CYP2D6 substrates [235]. Depending on the enzyme affected, this simplified scheme can be used as a basis for preliminary predictions of the effect a particular genotype will have on responsiveness to pharmacologic treatments. However, despite theoretic considerations of using genetic alteration in CYP enzymes, there has thus far been insufficient evidence for clinical applicability of CYP genotyping prior to prescribing either antidepressants (in particular, SSRIs) or antipsychotics for their respective applications [236-238].

### Role of genetic alteration in treatment response in major depressive disorder

The response to antidepressants in MDD has been reported to vary as a function of specific genetic alterations in certain candidate genes (comprehensively reviewed by Porcelli *et al*. [239]). Genes that have an effect on serotonin signaling have been a major focus of study for the pharmacogenomics of depression. The polymorphism in the 5′-promoter region of *5-HTT*, *5-HTTLPR*, has been shown reliably to influence not only susceptibility for developing MDD, but also response to antidepressant therapy. A number of studies have linked the *5-HTTLPR* long (L) allele with greater therapeutic response to SSRIs [234,240-252]. Other studies have indicated that the *5-HTTLPR* short (S) allele is associated with poorer or slower therapeutic response to SSRIs [250,251,253-258]. However, a number of discordant studies have appeared. In a population of Korean patients, Kim *et al*. [259] found that the *5-HTTLPR* S-allele conferred a greater therapeutic response to SSRIs. Yoshida *et al*. [260] found this same association in a population of Japanese patients and Lotrich *et al*. [261] also found this association in older patients. Moreover, a number of studies failed to show any relationship between *5-HTTLPR* genotypes and antidepressant response or side effects [262,263]. Such discrepant findings may be related to particular ethnic or age-specific populations, and these factors should also be considered when evaluating genetic response to antidepressants.

There is also evidence for a pharmacogenomic role for genetic polymorphisms in the serotonin receptors HTR1A and HTR2A, although there are inconsistencies in these findings as well. Some studies showed an association between the *HTR1A* rs6295-C/C genotype and increased therapeutic response to SSRIs in Chinese populations [246,264,265] but Kato *et al*. [266] found the alternate genotype, rs6295-G/G, to be associated with increased response to SSRIs. However, other groups have been unsuccessful in identifying a relationship between SSRIs and polymorphisms in *HTR1A*[267-269]. The data for *HTR2A* presents similarly, with many authors observing that particular genetic alterations, including the rs6311-G allele, the rs9316233-G allele, rs2224721, rs1923884, rs7997012, rs6313, rs799701, rs3125, rs1923882, and rs6314, are associated with increased response to SSRIs [269-273]. Murphy *et al*. [274] compared the SSRI paroxetine with the tetracyclic antidepressant mirtazapine in an 8-week double-blind randomized control trial, to identify potential genetic predictors of efficacy and side effects. This group characterized variation in *HTR2A* in 246 older patients with MDD undergoing the trial, and found that discontinuing use of paroxetine was significantly associated with the *HTR2A* rs6313-C allele in a dose-dependent manner. Moreover, carriers of the *HTR2A* rs6313-C/C genotype experienced more severe side effects than did those with the other genotypes. Notably, there was no significant influence of the *HTR2A* rs6313 genotypes on mirtazapine side effects [274]. Such findings stand in contrast with other groups, who did not observe an effect of *HTR2A* variation on antidepressant response [246,275-277].

Because HPA axis disruption has been associated with MDD susceptibility and pathogenesis, genetic targets within the HPA axis represent plausible candidates for differential antidepressant response. Notably, polymorphisms in the *FKBP5* gene have been associated both with increasing susceptibility to MDD (whose conferred susceptibility is further attenuated by environmental stress) and with differential response to antidepressant therapy [24,278-280]. In fact, Binder *et al*. [24] characterized a significantly faster response to SSRIs, TCAs, and mirtazapine in patients with MDD who were homozygous (TT) for the *FKBP5* marker rs1360780 compared with the heterozygotes (TC) and homozygote major allele carriers (CC) combined. In a pharmacogenetic association study of genetic variants in *CRHBP* and response to citalopram, Binder and colleagues [281] identified a SNP significantly associated with both remission and reduction of depressive symptoms in Hispanic and African-American outpatients with MDD.

Finally, the endogenous opioid system has been implicated in MDD, making the μ-opioid receptor, OPRM1, an attractive target for evaluation of antidepressant response variance secondary to genetic variance [282]. Garricock *et al*. [282] genotyped 53 separate SNPs in the *OPRM1* gene in 1,631 STAR*D samples, and found significant associations for polymorphic variation in *OPRM1* and citalopram response and remission. Moreover, the association between the commonly studied SNP rs1799971 and citalopram treatment outcome provided some evidence that this effect is influenced by ancestry groups. The authors expressed some caution in the interpretation of the findings, because even with the use of the same samples, none of the *OPRM1* variants met genome-wide significance, and the associations identified could reflect a placebo effect [282].

Of note, there are four published GWAS for SSRI antidepressant response, three of which stem from assessment of the samples involved in the STAR*D study and one from the Genome-Based Therapeutic Drugs for Depression (GENDEP). Despite the number of GWAS, only one marker in the Papilin (*PAPLN*) gene had a sufficiently robust association with antidepressant response (to suicidal ideation) to survive correction for multiple testing [270,283-285]. This highlights a benefit of the GWAS approach to pharmacogenomic inquiries; based on a candidate gene approach, *PAPLN*, which encodes a proteoglycan-sulfated glycoprotein, would probably have been overlooked. However, the paucity of significant findings also emphasizes the likely multifactorial nature of antidepressant response, not easily uncovered with current GWAS designs [286].

### Role of genetic alteration in treatment response in bipolar disorder

Similar to antidepressant response in MDD, numerous candidate genes have been evaluated for the effect of their genetic variation on BD therapy, most of which have focused on lithium [1]. Lithium response to variation in candidate genes has some notable associations including *5-HTTLPR*, *CLOCK*, *BDNF*, X-box binding protein 1 (*XBP1*), glycogen-synthase kinase 3 beta (*GSK3B*), breakpoint cluster region (*BCR*), cAMP responsive element binding proteins 1to 3 (*CREB 1*, *2*, and *3*) and neurotrophic tyrosine kinase receptor, type 2 (*NTRK2*) [287-294].

### Role of genetic alteration in treatment response in schizophrenia

Numerous studies have begun to pinpoint associations between genetic variants in some individuals with SZ and the tolerability and therapeutic efficacy of antipsychotic medications [295-298]. Tardive dyskinesia (TD) is an often debilitating motor disorder characterized by hyperkinetic, repetitive, and involuntary movements, which is experienced by approximately 30% of individuals with chronic SZ on long-term antipsychotic treatment (usually with first-generation antipsychotics) [299]. There are several studies providing evidence for genetic variation affecting susceptibility to TD in response to antipsychotic treatment. For example, polymorphisms near the DA receptor *D2* gene (*Taq1A*, minor allele, rs1800497) and in the DA receptor *D3* gene (*DRD3*-Ser9Gly, rs6280) have been linked with protection against TD and increased susceptibility for developing TD, respectively [300-302]. However, Woo and colleagues [303] were unable to find any association between the *DRD3* Ser9Gly in their study of 113 Korean patients with SZ. Moreover, data analysis from the NIMH Clinical Antipsychotic Trials of Intervention Effectiveness (CATIE), a double-blind study that compared the first-generation antipsychotic perphenazine with four second-generation antipsychotics, did not find evidence for association with TD for either polymorphism [304,305]. Other genetic variations positively associated with TD have been identified in the genes for the COMT catecholamine-degrading enzyme (Met158Val, Val allele), the manganese isoform of superoxide dismutase (*MnSOD*), a mitochondrial enzyme involved in oxidative metabolism (homozygote Ala genotype at rs4880, *MnSOD*-Ala9Val, with a protective effect for Val carriers) and the serotonergic receptor gene *5-HT2A* (C allele at promoter region T102C, rs6313 in older patients) [306-310]. However, conflicting reports have also been published for the *MnSOD* and *5-HT2A* variants [311,312].

First-generation antipsychotics are associated with a risk for TD, whereas several of the second-generation antipsychotics (and first-generation antipsychotic, chlorpromazine) can increase the risk for the metabolic syndrome and or isolated weight gain in nearly one-third of patients with SZ [313-315]. Studies of association of genetic variation with these side effects include associations with polymorphisms in the gene for the serotonin receptor subtype 2C (*HTR2C*) and in the gene for leptin (*LEP*), which is important for satiety and adipose regulation. These association studies have reported positive associations with metabolic syndrome (*HTR2C* intragenic polymorphism rs1414334-C for risperidone and clozapine), less weight gain with *HT2RC* SNPs (*HTR2C* −759 C→T and −697 G→C promoter polymorphisms, rs3813929-T and rs518147-C [316-318], respectively), and increased weight gain with the *LEP* promoter SNP −2548 A→G (G allele) ([317,319]). However, contradictory findings have also been published [320,321]. Furthermore, Opgen-Rhein *et al*. [322] were unable to replicate the *LEP* association with increased weight gain.

Agranulocytosis, or severe reduction in a particular class of white blood cells, is a serious side effect occurring in approximately 1% of individuals receiving treatment with the atypical antipsychotic clozapine. This property limits clozapine’s clinical use despite its clear therapeutic efficacy in refractory patients [323,324]. Evidence for genetic variants conferring resistance or increasing susceptibility to clozapine-induced agranulocytosis (CIA) is reflected in a number of studies, although the relatively low incidence of CIA has limited study sizes and effective replication studies. The human leukocyte antigen (HLA) system is comprised of a host of genes that are important in immune system modulation, and it has thus far produced the most promising genetic associations with CIA [325]. These associations include several genetic variants involving the *HLA-DQB1* locus, and have become incorporated into a commercially available test, although the clinical usefulness of this test has not been established, owing to its low sensitivity [325,326]. Additionally, a small GWAS provides preliminary confirmation of the HLA-CIA association [327].

In a double-blind study, the NIMH CATIE compared the first-generation antipsychotic perphenazine with four second-generation antipsychotics: olanzapine, risperidone, quetiapine, and ziprasidone [304]. The CATIE trial is a rich resource explored by researchers interested in determining why there were differential responses to antipsychotics. A 2009 analysis of data from the CATIE study was unable to find any significant association (following correction for multiple testing) between 2,769 potential SNPs and 21 pharmacogenetic phenotypes [328]. A GWAS, also drawing from CATIE data, analyzed 738 subjects with SZ for association between SNPs and antipsychotic response, and identified one significant locus at rs17390445 associated with improved SZ-positive symptoms to ziprasidone treatment, although the specific biological relevance of the locus is unclear [329].

Studies of the gene encoding a voltage-gated potassium channel, subtype H (*KCNH2*), have generated some discussion about the possibility of detecting likely responders to atypical antipsychotic therapy. Previous reports of SZ-*KCNH2* SNP associations, along with knowledge that antipsychotics bind to *KCNH2*-encoded channels, fueled inquiry of how risk alleles in *KCNH2* might predict treatment response in SZ [330,331]. Apud *et al*. [332] analyzed data on the relationship between various *KCNH2* genotypes and atypical antipsychotic treatment response from two sources: the 364 patients of the CATIE study and an NIMH cohort of 54 partially treatment-resistant patients with SZ enrolled in a double-blind, placebo-controlled, crossover study (antipsychotics included olanzapine, quetiapine, risperidone, ziprasidone, and aripiprazole). The authors found that individuals with a TT genotype for rs1036145 (T allele previously established as an SZ-risk allele) exhibited significantly greater symptom improvement than those with either the TC or CC-genotypes (*P* = 0.0395) [332]. Specifically, two groups of patients homozygous for rs1036145-TT from the NIMH cohort (those receiving placebo first and those receiving active drug first), showed greater change in positive symptom ratings on the Positive and Negative Syndrome Scale (PANSS) compared with their heterozygote and CC-genotype counterparts. Similarly, in the larger CATIE group, patients with the rs1036145-TT or rs3800779-TT genotypes also showed greater improvement in positive symptoms and general psychopathology than their counterparts (rs1036145-TC/CC or rs3800779-TC/CC, respectively). Furthermore, patients carrying the rs1036145-TT homozygous genotype were far more likely to remain on their medication than the homozygous non-risk genotype (rs1036145-CC) (hazard ratio = 0.208,*P* = 0.033) [332]. The similar relationships of atypical antipsychotic treatment response with *KCNH2* genotypes in two separate trials is encouraging evidence that genotyping may help guide therapeutic decisions in SZ.

Finally, meta-analyses on the *DA D2* and *D3* receptor polymorphisms have been conducted, seeking association with therapeutic response to antipsychotics [333,334]. Zhang *et al*. [333]carried out a meta-analysis of 10 studies to determine the relationship between the *DRD2* polymorphisms, *Taq1A* and –141C Ins/Del, and antipsychotic response. They were unable to detect any relationship between clinical response and the *Taq1A* polymorphism A1/A1 genotype and A1 carriers compared with A2/A2 genotype (pooled OR = 1.39, 95% CI 0.91 to 2.13, *P* = 0.13 and pooled OR = 1.30, 95% CI 0.92 to 1.84, *P* = 0.14, respectively), despite previous association findings of clinical response to haloperidol in acute psychosis, risperidone in first-episode antipsychotic-naïve patients with SZ, and aripiprazole in patients with acutely exacerbated SZ [333,335-337]. They did report a significant relationship between the -141C Del carriers compared with patients with the 141C Ins/Ins genotype: -141C Del carriers tended to have poorer therapeutic response to antipsychotics compared with 141C Ins/Ins carriers (pooled OR = 0.65, 95% CI 0.43 to 0.97, *P* = 0.03) [333]. Results for association of the *DRD3* polymorphism, Ser9Gly, with antipsychotic treatment response have been equivocal. An initial meta-analyses conducted by Jönsson *et al*. [338] found that people with the *DRD3* Ser9 allele, the Ser/Ser and Ser/Gly genotypes were more likely to respond to typical antipsychotics, and less likely to respond to clozapine compared with their counterparts. However, a more recent meta-analysis by Hwang *et al*. [334] was unable to identify any significant association between the Ser9Gly polymorphism and antipsychotic drug response after increasing the sample size from 233 to 758 by including original analyses and studies published after the Jönsson meta-analysis.

## Emerging applications

The types of applications that will greatly affect the prospect of personalized medicine in psychiatry will involve continued improvements in genomic inquiries and continued development of genomic testing protocols. Apart from increasing efficiency in genomic techniques, expanding the substrate types for study to include transcriptomics and proteomics (the study of all expressed messenger RNA (mRNA) and all proteins, respectively), will exponentially improve the knowledge base and ability to make informed decisions in personalizing psychiatry [339]. Furthermore, studies that take a multidisciplinary approach, combining the categories (for example, gene × environment interactions or endophenotypes × environmental interactions.) that generate unique phenotypic expression of illness will provide new perspectives and will increase the database from which to draw further studies. Moreover, development of tools to manage and integrate this information and the development of pharmacogenomic tests for use in clinical practice will be greatly beneficial in generating clinically relevant decision trees for use in personalized psychiatry [340]. For example, targeting the molecules responsible for epigenetic regulation may prove to be a promising means of modifying outcomes in psychiatric illnesses [134]. In addition, studies that relate genetics not only to pharmacologic therapy, but to other psychotherapeutics, including various psychotherapies (such as cognitive behavioral therapy and dialectical behavioral therapy), light therapy, deep-brain stimulation, electroconvulsive therapy, and transcranial magnetic stimulation (TMS), will serve to further refine and optimize psychiatric care. Finally, the importance of developing neuroimaging methods and their application to psychiatry will continue to have significant effects on personalizing psychiatry, and this is discussed in greater detail below.

### Imaging genetics in psychiatry

A rapidly advancing area likely to have a major influence on personalized medicine in psychiatry is neuroimaging, and more specifically, neuroimaging genomics. Briefly, imaging genetics uses imaging measures as quantitative biological phenotypes. It seeks to incorporate genetics, psychiatry, and neuroscience in a way that relates genetic variation with protein function, brain structure and connectivity, and psychopathology [341]. Neuroimaging genetics allows conduction of experiments that relate genetic change to outcomes of measurable, repeatable tests of brain structure or function. Two major neuroimaging genetic approaches are 1) identification of imaging alterations in a population with a well-defined genetically determined illness; and/or 2) verification of the effects of specific genetic changes. A particularly useful aspect of defining biological phenotypes using imaging is the distancing of disease from subjective self-report and inconsistent diagnostics [341]. Furthermore, the ability to study *in vivo* change and change over time in psychiatric illness is paramount given the neuronal plasticity (or lack thereof) already identified in various psychopathologies. In essence, neuroimaging genetics has the potential to lead to better comprehension of the pathophysiology of common psychiatric disorders by evaluating the relationship between imaging phenotypes and genetic variation. Once interventions such as antidepressant therapy or TMS treatment (discussed below) are introduced into the equation, conclusions drawn from correlations may give rise to an understanding of causation. Explorations of concurrent intervention with real-time imaging, such as the assessment of self-regulation of frontal cortical activation (using neurofeedback) in nicotine dependence and craving, coupled with real-time fMRI, have led to this technology and its applications expanding in exciting new directions [342]. For example, trials using this type of volitional reduction with neurofeedback in the treatment of MDD are under way, building upon previous works by the same group ([343]; I. Gotlib, personal communication, 2012). One group is conducting additional trials in PTSD using concurrent TMS-EEG (technique as described by Johnson *et al*. [344]), which employs active therapeutic intervention with concurrent imaging (A. Etkin, personal communication, 2012) and is probably informed by the group member’s previous characterization of anxiety using fMRI [345].

Furthermore, there already exist a number of examples of imaging genetics with psychiatric implications [341]. We describe several examples that epitomize the types of studies and progress being made in imaging genetics (additional notable findings relevant to genes discussed throughout the review can be found in Table 1). Prominent examples are seen with in MDD, with the association between smaller hippocampal volumes and polymorphisms within the *5-HTTLPR* region, and with the *BDNF* Val66Met polymorphism [346]. However, there are some limitations to this field. Neuroimaging genetics is a relatively new area, subject to initial missteps, including false positives and imprecise interpretations. There is skepticism that functional neuroimaging studies (for example, those involving fMRI) actually show the functional change being queried. The output measure of fMRI is blood oxygen level dependent (BOLD) change, which is thought to reflect neuronal activation. Of note, there is a significant lag between the supposed measured neuronal activity and fMRI change due to the cardiovascular changes necessary to increase BOLD in response to neuronal activation. Various imaging methods, each with their own strengths and weaknesses, have the potential to contribute to clarification of this process, often in complementary ways. For example, where there is some temporal disconnection between neuronal activation and BOLD response, diffusion tensor imaging (DTI), which is sensitive to directional diffusion of water along neural pathways, exhibits a more proximate relationship to neuronal activation [347]. MR spectroscopy (MRS) can provide chemical profiles capable of differentiating between brain pathologies. Although the use of positron emission tomography/single-photon emission computed tomography (PET/SPECT) is more invasive than the other methods described, it has the advantage of allowing *in vivo* monitoring of molecular changes such as receptor or transporter binding density in response to therapy [348-350] (a summary of select imaging findings is given in Table 1).

**Table 1:** Selection of imaging findings in psychiatric illness

**Psychiatric illness**	**Subgroup/genotype**	**Imaging feature**	**Sample**	**Imaging method**	**Reference**
Major depressive disorder		• Reduced activity in frontal lobes	Various	PET, fMRI, EEG, SPECT	[351]
		• Reduced HPC volumes	Meta-analyses	MRI	[352,353]
		• Greater reduction in HPC volume with increased duration of untreated MDD	38 male outpatients	MRI	[354]
		• Increased baseline activity in pulvinar nuclei bilaterally	Meta-analysis	PET and SPECT	[343]
		• Increased amygdala, dorsal ACC, and insular response to negative stimuli compared with healthy controls	Meta-analysis	Task-based fMRI	[343]
		• Reduced dlPFC and dorsal striatum response to negative stimuli compared with healthy controls	Meta-analysis	Task-based fMRI	[343]
	5-HTTLPR -S or Lg allele:	Increased amygdala reactivity to masked emotional faces; correlated to lifetime psychiatric hospitalization in MDD	35MDD, 32 controls	fMRI	[355]
	5-HTTLPR -S or Lg allele:	Increased bilateral amygdala activation after emotional stimuli (allele effects are additive)	27 MDD on medication	fMRI	[356]
	5-HT1A - 1019G allele:				
	5-HTTLPR- L/L:	Reduced HPC volumes compared with controls	40 MDD, 40 controls	MRI	[357]
Late onset (LO) MDD	5-HTTLPR- L/L:	Reduced HPC volumes compared with S/L or S/S genotypes	63 LO MDD, 72 EO MDD, 83 controls	MRI	[358]
Early onset (EO) MDD	5-HTTLPR -S allele:	Reduced HPC volumes compared with left allele carriers			
Bipolar disorder		• Increased size of lateral ventricles (right ventricle only in [359])	Meta-analyses	CT and MRI	[359-361]
		• Increased number of deep white-matter hyperintensities	Meta-analyses	CT and MRI	[360-363]
		• Increased number of subcortical gray-matter hyperintensities	Meta-analysis	MRI	[362]
		• Increased activity in limbic structures (left side only in [364])	Meta-analyses	fMRI and PET	[364,365]
		• Reduced activity in frontal structures (vlPFC and dlPFC [364])			
	Pediatric population:	• Reduced amygdala volume	Meta-analyses	MRI	[365,366]
Schizophrenia		• Reduced frontal-lobe activity at rest and during task activation	Meta-analysis	MRI, PET	[367,368]
		• Increased lateral ventricular size	Meta-analyses	CT and MRI	[369,370]
		• Increased D2 dopamine receptor density	Meta-analysis	PET and SPECT	[371]
		• Reduced frontal gray matter	Meta-analyses	MRI	[372,373]
	SZ:	• Reduced bilateral HPC volume relative to controls	Meta-analyses	MRI	[374-376]
	nonpsychotic 1^st^ deg family:		Meta-analysis	MRI	[370]
	BDNF 66Val/Val carriers:	• Reduced activation of the cingulate, lateral PFC and lateral parietal regions during verbal memory task	58 high-risk subjects (first-degree or second-degree family with SZ)	fMRI	[377]

+/−A, alcohol abuse positive/negative subjects; ACC, anterior cingulate cortex; Cho, choline; CT, computed tomography; DA, dopamine; dlPFC, dorsolateral pre-frontal cortex; EO, early onset; fMRI, functional magnetic resonance imaging; ^1^H-MRS, proton MR spectroscopy; HPC, hippocampal; LO, late onset; MDD, major depressive disorder; MRI, magnetic resonance imaging; NAA, *N*-acetylaspartate; parahippocampal gyrus; PET, positron emission tomography; Tx, treated; vlPFC, ventral-lateral pre-frontal cortex; vmPFC, ventral-medial pre-frontal cortex.

## Conclusion

In this review, we have focused on the components and tools that are proving to be instrumental in personalizing medicine in psychiatry. We have discussed the types of information that can be garnered and eventually used in tailoring psychiatric therapies to the individual. This information can be found in the form of particular genetic and epigenetic changes more characteristic in the psychiatrically ill or in observable biomarkers that are reflective of illness. Additionally, environmental influences are evaluated; in particular, how their interaction with genetic variation can lead to disease attenuation or exacerbation. Furthermore, we have discussed how the definition of the illnesses can influence the tailoring of individualized therapies. For example, many psychiatric disorders show phenotypic heterogeneity at the same time as having symptoms that overlap with other psychiatric illnesses that may presumably share some fundamental biologic underpinnings. Indeed, uncovering the biological basis of individual symptoms may prove to be as or more helpful in understanding the pathophysiology of the illness than forcing a constellation of co-occurring symptoms to fit together under one biologically plausible explanation. What we will probably experience is a refining of the diagnostic process; in some cases, recognizing spectrums of disease and in others, homing in on specific biological features of an illness.

It should be noted that these categories for defining an individual’s unique psychiatric phenotype are artificially separated to facilitate a conceptual framework, and there is substantial overlap between each category (Table 2). For example, an illustration of how genetic variation interacts with environmental factors is apparent in *CRHR1* polymorphism haplotypes, which are not only associated with MDD but also interact synergistically with childhood trauma to increase the risk of MDD [217,281]. Another example is the dexamethasone-binding capacity of leukocytes; although this is not used in the diagnosis of PTSD, it can be used as a biomarker or a proxy measure for glucocorticoid receptor number. In turn, this measurement might help screen persons likely to develop PTSD, because greater glucocorticoid receptor density is predictive of risk for PTSD symptoms in military personnel returning from deployment [378].

**Table 2:** Psychiatric disease susceptibilities

**Gene(s)**	**Variant(s)**	**Population(s)**	**EPI**	**EF**	**BM**	**Key findings**	**Outcome**	**Ref.**
Major depressive disorder								
*5-HTT*	*5-HTTLPR* and EF: stressful life events	Caucasian		+		Homozygous or heterozygous carriers of the short allele had higher frequency of depression and suicidality when exposed to stressful life events.	SIG	[221]
*5-HTT*	STin2.9	Caucasian				Increased frequency in MDD relative to controls	SIG	[7]
*5-HTT*	*5-HTTLPR*	Caucasian				Increased frequency in MDD relative to controls	SIG	[8]
*TPH1*	microsatellite at 11p15.3-p14	Caucasian community-based sibships				Association with MDD susceptibility and microsatellite	SIG	[11]
*TPH1*	Various	Caucasian				Six haplotypes associated with MDD risk	SIG	[12]
*TPH2*	rs120074175 (p.R441H)	Caucasian (90%), AA (8%), East Asian (2%)				Higher frequency of SNPs in patients with MDD compared with controls or patients with BP	SIG	[15]
*TPH2*	rs120074175 (p.R441H)	Caucasian (84%), Hispanic (6%), East Asian (5%), AA (3%), others (2%)				SNP not identified in non-treatment-resistant and treatment-resistant patients with MDD, or in treatment-resistant patients with BP, or in controls	NS	[16]
*TPH2*	rs120074175 (p.R441H), rs1843809 (c.608 + 5263G>T)	Caucasian				Higher frequency of SNPs in MDD relative to controls	SIG	[13]
*TPH2*	Various	East Asian (Korean)				No association of the SNPs rs4570625, rs10748185, rs11179027, rs4469933, or rs17110747 in MDD, BP, or SZ	NS	[17]
*TPH2*	rs4570625 (c.-141-703G>T), rs17110747 (c.*479G>A)	Meta-analysis				SNPs associated with MDD susceptibility by fixed-effects modeling; rs4570625 remained significant using random-effects calculations	SIG	[9]
*TPH2*	rs4570625-rs10748185 (G>A).	East Asian (Korean) inpatients				Haplotype significantly associated with higher MADRS endpoints in MDD	SIG	[19]
*FKBP5*	rs3800373 (c.*1136G>T)-(CC) rs1360780 (c.106-2636A>G)	Post-mortem brain samples, ethnicity not specified				Five clinical groups were compared: MDD, MDD + psychosis, MDD + HIV, HIV-positive, and HIV-negative. Genotype frequencies in the MDD and the MDD + psychosis groups differed from published allelic frequencies	SIG	[25]
*FKBP5*	rs1360780 (c.106-2636A>G)	Caucasian inpatients with MDD, BD, or dysthymia				Carriers of the TT genotype experienced more depressive episodes, by a factor of 2:1 compared with the CC or CT genotypes	SIG	[24]
*FKBP5*	rs1360780 (c.106-2636A>G) (TT), rs3800373 (GG)	Caucasian treatment-resistant adolescents				Genotypes were associated with suicidal events	SIG	[26]
*FKBP5*	rs9470080, rs9394309, rs7748266, rs1360780; BM: reduced daytime cortisol secretion	Caucasian older people			+	Minor alleles were associated with decreased daytime cortisol levels and increased likelihood of depressive symptoms	SIG	[279]
*FKBP5*	rs9470080 (c.-19-35815A>G), rs9296158 (c.509-1901T>C) and EF: prolonged stress exposure	East Asian (Korean)		+		Two SNPs were associated with anxiety and depression after prolonged stress in patients with cancer patients	SIG	[280]
*CRHR1*	rs110402 (GG), rs242924 (GG); and EF: childhood trauma; and BM: response to DEX/CRH test	Healthy Caucasians with history of early life stress		+	+	In adults who had experienced maltreatment, the GG genotypes were associated with increased cortisol response to DEX/CRH test	SIG	[219]
*CRHR1*	rs10473984 EF: childhood trauma			+		SNP works synergistically with childhood trauma to increase risk of MDD	SIG	
*CRHR1*	rs110402 (c.34-4338G>A); EF: childhood abuse; and BM: cortisol response to DEX/CRH test	1: AA, 2: ethnically diverse		+	+	In adult men who had experienced child abuse, the A allele was associated with reduced MDD symptoms and reduced cortisol response to DEX/CRH test	SIG	[220]
*CRHR1*	rs110402 (c.34-4338G>A), rs7209436 (c.33 + 8207C>T) and rs7209436-rs110402-rs242924 (TAT); EF: childhood abuse	AA, Caucasian		+		Rare alleles were protective in a dose-dependent manner against MDD in the presence of child abuse	SIG	[217]
*CRHR1*	rs7209436-rs110402-rs242924 (TAT); EF: childhood abuse	Caucasian (>90%)		+		TAT haplotype was protective against MDD in women exposed to severe maltreatment, but not in a replication study using different measure of trauma	SIG	[218]
*CRHR1*	rs242939 (c.241 + 1631C>T), three haplotypes	East Asian (Chinese)				Allele and genotype association with MDD	SIG	[32]
*CRHR1*	rs110402 (c.34-4348G>A)	Caucasian				Association between SNP and early onset of MDD and increased risk for a seasonal pattern	SIG	[33]
*CRHPB*	Haplotype block	Caucasian (Swedish)				In patients with recurrent MDD, haplotype block (s02-TT and s11-TT and s14-T) was significantly associated with disease compared with controls	SIG	[35]
*CRHPB*	Haplotype block	Caucasian (Swedish and Belgian)				Could not replicate findings of [35] in an extended Swedish or Belgian sample. Found higher frequency of haplotype block (s02-TT, s11-TT and s12C) in Swedish men compared with control men	NS	[36]
*HTR3A*	42 (CC); EF: early life stress (ELS); BM: frontolimbic gray-matter alterations	Healthy Caucasian		+	+	Genotype + ELS was a predictor of depressed mood. Carriers had greater frontolimbic gray-matter alterations, which were increased by ELS	SIG	[379]
*SYNE1*	rs9371601 (c.1653 + 2159C>A)	Caucasian				Higher frequency of SNPs in recurrent MDD relative to controls	SIG	[52]
*NR3C1*	EPI: *NR3C1* promoter site methylation; and EF: history of childhood abuse	Suicide victims	+	+		In abused victims, *NR3C1* promoter methylation was increased and glucocorticoid receptor mRNA reduced compared with non-abused victims or controls	SIG	[144]
--	BM: CSF concentration of CRF	Various			+	Increased CSF concentration of CRF is a replicable finding in MDD. Also seen in suicide victims	SIG	[28-31]
--	EF: birth trauma	Monozygotic twins discordant for MDD		+		Increased occurrence of birth trauma in SZ-affected twin	SIG	[223]
--	EF: obstetric complications, e.g. abnormal fetal growth/development, pregnancy and delivery complications	Meta-analysis of population-based prospective studies		+		Obstetric complications increased risk for SZ	SIG	[224]
--	BM: CSF concentration of norepinephrine metabolite MHPG	Caucasian (81%) with MDD (85%) or BD (15%)			+	Lower levels of MHPG were predictive of suicidal behavior, and correlated with higher medical lethality of suicide attempt	SIG	[190]
--	rs1360780 (c.106-2636A>G)	Caucasian, Black				Association of SNP with MDD risk in Caucasian sample	SIG	[34]
Bipolar disorder								
*FKBP5*	rs4713902 (c.-19-3406A>G), rs7757037 (c.841-238C>A), rs9296158 (c.509-1901T>C), rs3800373 (c.*1136G>T), rs9380525 (c.-19-22418C>G)	Family trios and quads with BD-I, or BD-II + rMDD, or SZA-BD				SNPs associated with BD in populations studied (BD-I, BD-II + rMDD, SZA/BD); rs4713902 remained significant after correction for multiple testing	SIG	[40]
*FKBP5*	various	Caucasian (Ashkenazi Jewish)				No significant SNP or haplotype associations with BD or SZ identified	NS	[41]
*FKBP5*	rs4713916 (c.20 + 18122T>C), rs1360780 (c.106-2636A>G), rs380037	Caucasian				No significant association between SNPs and BD	NS	[42]
*ARNTL*	rs7107287 (c.-208 + 13499G>T), rs895682 (c.-135 + 13626T>C), rs1481892 (c.-208 + 2451G>C), rs4757142 (c.-207-5839G>A)	Caucasian family trios				SNPs rs7107287 and rs895682 showed significant transmission bias in family samples. In Pittsburg sample, genotype distribution of SNPs rs1481892, rs7107287 and rs4757142 differed from that of controls	SIG	[48]
*TIMELESS*	rs2279665 (c.114G>C), rs2291738 (c.2726-4A>G), rs774026 (c.1578 + 22T>C), rs2291739 (p.P1018L)	Caucasian family trios				SNPs (rs2279665, 2291738) showed transmission bias in family samples. Haplotype over-transmission involving SNPs rs2279665, rs774026, rs2291738, and rs2291739	SIG	[48]
*CLOCK*	rs534654 (c.793-485A>G), rs6850524 (c.-289-5765G>C), rs4340844 (c.559 + 996T>G)	Family trios and quads				Suggestive evidence for transmission disequilibrium	SUG	[46]
*SYNE1*	rs9371601 (c.1653 + 2159C>A)	Caucasian				Higher frequency of SNP in BD compared with controls	SIG	[52]
*COMT*	EPI: *MB-COMT* promoter methylation	Post-mortem brain samples (97% Caucasian)	+			Reduced methylation of *COMT* promoter in BD compared with controls led to higher MB-COMT expression in BD compared with controls	SIG	
*COMT*	EPI: *MB-COMT* promoter methylation	Caucasian post-mortem brain samples	+			Promoter methylation did not differ between BD and control brains	NS	[151]
--	EF: obstetric complications	Meta-analysis		+		No findings to suggest higher risk for BD relative to MDD or controls after exposure to obstetric complications	NS	[225]
--	BM: peripheral blood levels of BDNF	Meta-analysis			+	Relative to controls, patients with BD in manic or depressed states had reduced serum and plasma BDNF levels	SIG	[197]
--	BM: serum or plasma levels of BDNF	Meta-analysis			+	Relative to controls, patients with BD in manic or depressed states had reduced serum and plasma BDNF levels	SIG	[196]
Schizophrenia								
GWAS	various	GWAS of MGS sample (Caucasian, AA)				No significant finding in MGS case–control sample GWAS	NS	[58]
MHC region on chr6	rs3130375 (7kb from *NOTCH4*) and large sets of nominally associated ‘score alleles’	Caucasian, AA				Imputed SNP rs3130375 reached genome-wide significance. Strong suggestion for a polygenic basis for SZ	SIG	[95,96]
MHC region on chr6	various	Meta-analysis of MGS, ISC, and SGENE data				Association between SZ and region of LD on chromosome 6p22.1	SIG	[58]
MHC region on chr6	*HIST1H2BJ*: rs6913660, *PRSS16*: rs13219354, rs6932590, *PGBD1*: rs13211507 (c.642 + 2432T>C), *NOTCH4*: rs3131296 (c.2866-827A>G)	GWAS of SGENE-plus, ISC, and MGS (Caucasian)				With combined samples, MHC region SNPs showed genome-wide significance	SIG	[59]
*COMT*	rs165688 (p.V158M)	Caucasian with velocardiofacial syndrome (VCFS) ± SZ				No correlation between allelic distribution and SZ in individuals with VCFS	NS	[105,380]
*COMT*	rs165599 (c.*522G>A), rs737865 (c.-92 + 701A>G), rs165688 (p.V158M)	Caucasian (Ashkenazi Jewish)				G allele in the SNPs was associated with SZ. Haplotype rs737865-rs165599 (G-G) had most significant overall association with SZ	SIG	[106]
*COMT*	rs737865 (c.-92 + 701A>G)	Meta-analysis (Caucasian)				Nominally significant association between SNP and SZ in analyses restricted to European samples	SIG	[82]
*DISC1*	t(1:11)(q43,q21)	Caucasian (Scottish pedigree)				Translocation found to be in significant LD with SZ	SIG	[107,108]
*DISC1*	rs821616 (p.S704C), rs821597 (c.2042 + 7630G>A), rs7546310 (c.1982-32754A>C) BM: hippocampal structure and function	Caucasian, replication: family trios (Caucasian and AA)			+	704-Ser associated with altered hippocampal structure and formation in healthy subjects. Association between 704-Ser and SZ. Three-SNP haplotype associated with SZ in the family sample	SIG	[112]
*DISC2*	n.9481C>T, n.11085C>A, n.11160G>A, n.11870T>C, n.11859T>C	Caucasian (Scottish)				No co-segregation with SZ or BD or significant association was detected. SNPs were not in LD	NS	[132]
*COMT*	EPI: Membrane-bound *COMT* (*MB-COMT*) promoter methylation	Caucasian post-mortem brain samples	+			*COMT* promoter methylation did not differ between SZ and control brains	NS	[151]
*COMT*	*MB-COMT* promoter EPI: *COMT* methylation.	Post-mortem brain samples (97% Caucasian)	+			Reduced methylation of *COMT* promoter in SZ compared with controls, resulting in increased MB-COMT expression in SZ compared with controls	SIG	[148]
*ZNF804A*	rs1344706 (c.256-19902A>C)	GWAS: Caucasian (English); replication: Caucasian and East Asian (BUL, GRM, US, AUS, JPN, CHN, and ISR)				Nominally significant association between SNP and SZ in samples; genome-wide association when case sample extended to include BD	SIG	[60]
*ZNF804A*	rs1344706 (c.256-19902A>C), rs7597593 (c.111 + 69783T>C), rs17508595 (c.111 + 19311C>G)	Caucasian (Irish)				Nominally significant association between SNPs and SZ + poor-outcome schizoaffective disorder	SIG	[61]
*ZNF804A*	rs12477914 and rs1366840 as surrogates for rs1344706 (c.256-19902A>C)	Initial study: Caucasian; follow-up: Caucasian + CHN				Nominally significant association between SNPs and SZ. When stratified by population, significant in 2 (RUS and DNK) of 13 (HUN, NOR, RUS, SWE, FIN, DEU, DNK, GBR, SCO, ISL, NLD, ITA, CHN) ethnic groups	SIG	[62]
*ZNF804A*	rs1344706 (c.256-19902A>C)	East Asian (Han Chinese)				Nominally significant association between SNP and SZ in a population-based sample. In a family-based trio study, trend toward significant over-transmission	SIG/SUG	[63]
*TCF4*	rs9960767 (c.146-23634T>G)	Caucasian (BEL, DNK, DEU, IRL, ITA, FIN, SPA, UK, USA)				Association between the C allele and SZ in GWAS and in replication studies	SIG	[59,69]
*TCF4*	rs2958182 (c.146-17653T>A) (as surrogate for rs9960767)	East Asian (Han Chinese)				SNP substituted for rs9960767 as rs9960767 is not polymorphic in CHN, is in LD with rs9960767, and is significantly associated with SZ in CHN	SIG	[71]
*TCF4*	rs12966547 (g.542881G>A)	Caucasian				Significant association between SNP and SZ	SIG	[70]
*NRG1*	HapICE (SNP8NRG221132, SNP8NRG221533, SNP8NRG241930, SNP8NRG243177 and SNP8NRG433E1006, & microsatellite repeats 478B14-848 and 420M9-1395)	Caucasian				Haplotype significantly associated with SZ, with a relative risk of 2.2	SIG	[77]
*RELN*	EPI: *RELN* promoter methylation	Post-mortem brain samples	+			Increased methylation of *RELN* promoter in SZ compared with controls, leading to reduced RELN mRNA expression	SIG	[150]
*RELN*	EPI: *RELN* promoter methylation	Post-mortem brain samples	+			By contrast to [150], neither SZ nor control samples found promoter hypermethylation	NS	[152]
*HTR2A*	EPI: cytosine methylation at rs6313 (c.102>T)	Post-mortem brain samples	+			102C carriers have reduced *5HT2A* gene expression. In SZ, there is a greater reduction in carriers than in non-SZ carriers. Antipsychotics that reduce CpG methylation lead to increased HTR2A expression	SIG	rev. in [153]
*TPH2*	rs4570625 (c.-141-703G>T) rs4570625- rs4565946 ((c.-141-703G>T)-(c.255 + 1256C>T) (G-C))	Caucasian				Higher frequency of SNP in patients with MDD compared with controls in discovery sample; not replicated in replication sample. Trend for rs4570625-rs4565946 G-C haplotype	SUG	[18]
*KCNH2*	rs1036145 (c.76 + 496G>A)	NIMH and CATIE cohorts				Carriers of rs1036145-TT genotype showed greater change on the PANSS than carriers of TC and CC genotypes. rs1036145-TT and rs3800779-TT showed significant improvement in positive symptoms compared with TC/CC genotypes	SIG	[332]
--	EF: prenatal exposure to influenza (determined by ecologic data only)	Caucasian (Finnish)		+		Exposure to influenza during second and third trimesters increased risk of hospitalization for SZ	SUG	[226]
--	EF: prenatal exposure to influenza (determined by ecologic data only)	Caucasian (English, Welsh)		+		Number of births with subsequent SZ development was higher during influenza epidemic relative to corresponding time during non-epidemic years	SUG	[227]
--	EF: prenatal exposure to influenza (serologically documented)	Caucasian, AA, Others (Native American, MEX, East Asian)		+		Early to mid-gestational exposure to influenza increased risk for SZ	SIG	[228]
--	EF: prenatal exposure to influenza	Meta-analysis		+		No association between exposure and SZ identified	NS	[229]
--	EF: prenatal exposure to maternal stress (wars, spousal demise, disasters, etc.)	Meta-analysis		+		Data show no effect of prenatal stress on risk for SZ	NS	[230]

5-HTTLPR, 5-hydroxytryptophan transporter-linked polymorphic region; AA, African-American; AU, Australian; BD, bipolar disorder; BD-I, BD-II bipolar disorder types I and II; BDNF, brain-derived neurotrophic factor; BEL, Belgian; BGR, Bulgarian; BM: biomarkers; CATIE, Clinical Antipsychotic Trials of Intervention Effectiveness; CHN, Chinese; CRF, corticotropin-releasing factor; CSF, cerebrospinal fluid; DEX/CRH, Dexamethasone/corticotropin-releasing hormone; DNK, Danish; EF, environmental factors, ELS, early life stress; EPI, epigenetic factors; FIN, Finnish; GRM, German; GWAS, GWAS, Genome-wide association studies; HIV, human immunodeficiency virus; ISL, Icelandic; IRL, Irish; ISC, International Schizophrenia Consortium; ITA, Italian; ISR, Israeli; JPN, Japanese; KOR, Korean; LD, linkage disequilibrium; MB-COMT, membrane-bound catechol-O-methyltransferase; MDD, major depressive disorder; MEX, Mexican; MHPG, 3-methoxy-4-hydroxyphenylglycol;NIMH, National Institute of Mental Health; NLD, Dutch (Netherlands); NS, not significant; rMDD, recurrent MDD; RUS, Russian; SCO, Scottish; SGENE, Schizophrenia Genetics Consortium; SIG, significant; SNP, single-nucleotide polymorphism; SPA, Spanish; SUG, suggestive; SZ, schizophrenia; SZA-BD, schizoaffective disorder, manic or bipolar type; UK, United Kingdom; USA, American; VCFS, velocardiofacial syndrome.

We also discussed the use of pharmacogenomics in psychiatry. The best-studied pharmacogenomics in psychiatry are the CYP450 liver enzymes, responsible for the metabolism of many psychotropic drugs. Various polymorphisms in these enzymes predispose an individual to enhanced or poorer therapeutic and/or side-effect response to certain medications. Despite robust findings of association with the particular CYP450 genotypes and altered response to psychotropics, there remains insufficient evidence to support CYP450 genotype screening [236,381]. Additionally, many of the genetic alterations described in this review also are relevant to pharmacogenomic paradigms. Examples include the poor response to SSRIs seen in Caucasian women with MDD who are carriers of the *5-HTTLPR* short allele, or the faster response to SSRIs seen in patients with MDD who are homozygous T for the *FKBP5* marker rs1360780 [24,253]. Naturally, there is still work to be done in psychiatric pharmacogenomics, as causative treatment strategies for these disorders have yet to be implemented.

The prospect of personalized medicine in psychiatry more or less reflects ideals still largely unrealized. Currently, the field is at the information-gathering infancy stage. The greatest progress can be expected at the intersections of the categories described above, such as gene × environment and genes × biomarkers, which will poise psychiatry to make biological system-based evaluations. Furthermore, some of the emerging applications, including imaging genomics, strengthen our conviction that the future for personalized medicine is highly promising.

## Abbreviations

5-HT: 5-hydroxytryptophan (serotonin); 5-HTTLPR: 5-HT transporter-linked polymorphic region; ACC: anterior cingulate cortex; ANK3: ankyrin 3; APOE: apolipoprotein E; ARNTL: aryl hydrocarbon receptor nuclear translocator-like BmaL1; BCR: breakpoint cluster region; BD: bipolar disorder; BDNF: brain-derived neurotrophic factor; BOLD: blood oxygen level dependent; CACNA1C: calcium channel, voltage-dependent, L type alpha 1C subunit; CATIE: Clinical Antipsychotic Trials of Intervention Effectiveness; Cho: choline; CIA: clozapine-induced agranulocytosis; CLOCK: circadian locomotor output cycles kaput protein; CNS: central nervous system; COMT: catechol-O-methyltransferase; CpG: cytosine-phosphate-guanine; CREB: cAMP responsive element binding proteins 1 to 3; CRF: corticotropin-releasing factor; CRH: corticotropin-releasing hormone; CRHBP: corticotropin-releasing hormone-binding protein; CRHR1: corticotropin-releasing hormone, receptor 1; CRY1/CRY2: cryptochrome 1 and 2; CSF: cerebrospinal fluid; CT: computed tomography; CYP: cytochrome P450; DA: dopamine; DAO: D-amino acid oxidase; DEX/CRH: dexamethasone/corticotropin-releasing hormone; DISC1: disrupted in schizophrenia, 1; dlPFC: dorsolateral pre-frontal cortex; DME: drug-metabolizing enzymes; DMNT: DNA methyltransferase; DRD1/2/4: dopamine receptor, D1/D2/D4; DTI: diffusion tensor imaging; DTNBP1: dystobrevin binding protein 1; ECT: electroconvulsive therapy; EM: extensive metabolizers; FKBP5: FK506-binding protein; fMRI: functional magnetic resonance imaging; GABA: gamma-aminobutyric acid; GABRB2: GABA A receptor, beta 2; GENDEP: Genome-Based Therapeutic Drugs for Depression; GHQ: General Health Questionnaire; GRIN2B: glutamate receptor, ionotrophic, N-methyl D-aspartate 2B; GWAS: genome-wide association studies; 1H-MRS: positron magnetic resonance spectroscopy; ISC: International Schizophrenia Consortium; HDAC1: histone deacetylase 1; HDACi: HDAC inhibitors; HLA: human leukocyte antigen; HP: haptoglobin; HPA: hypothalamic-pituitary-adrenal; HPC: hippocampus; HTR2A and HRT2C: 5-hydroxytryptamine (serotonin) receptor 2A and 2C, G-protein coupled; IL-1B: interleukin 1 beta; KCNH2: potassium voltage-gated channel, subfamily H; IM: intermediate metabolizers; ISC: International Schizophrenia Consortium; LEP: leptin; LD: linkage disequilibrium; LG: licking and grooming; LINC: links the nucleoskeleton to the cytoskeleton; MAOA: monoamine oxidase A; MB: membrane-bound; MCM: mood-congruent memory; MDD: major depressive disorder; MGS: Molecular Genetics of Schizophrenia; MHC: major histocompatibility complex; MHPG: 3-methoxy-4-hydroxphenylglycol; miRNA: microRNA; MnSOD: manganese isoform of superoxide dismutase; MRI: magnetic resonance imaging; MRS: mRNA, messenger RNA; MRS: magnetic resonance spectroscopy; MTHFR: methylenetetrahydrofolate reductase (NAD(P)H); NAA: *N*-acetylaspartate; NCAN: neurocan; ND: never-depressed; NIMH: National Institute of Mental Health; NIMH-ECA: National Institute of Mental Health Catchment Area; NMDA: N-methyl-D-aspartate; NR2B: NMDA receptor subunit 2B; NR3C1: neuron-specific glucocorticoid receptor; NRG1: neuregulin 1; ODZ4: odd Oz/ten-m homolog 4; PANSS: Positive and Negative Syndrome Scale; PAPLN: papilin; PER1/PER2/PER3: period 1/2/3; PET: positron emission tomography; PFC: pre-frontal cortex; PLXNA2: plexin A2; SLC6A4: dopamine transporter gene A4; PM: poor metabolizers; PTSD: post-traumatic stress disorder; RD: remitted-depressed; SGENE: Schizophrenia Genetics Consortium; sib-tbt: Sibling-Transmission Disequilibrium Test; SNP: single-nucleotide polymorphism; SSL: schizophrenia susceptibility locus; SSRI: Selective serotonin reuptake inhibitor; STAR*D: Sequenced Treatment Alternatives to Relieve Depression; SYNE: spectrin repeat containing, nuclear envelope; SZ: Schizophrenia; SPECT: single-photon emission computed tomography; TP53: tumor protein p53; TPH 1/2: tryptophan hydroxylase 1/2; TD: tardive dyskinesia; TORDIA: Treatment of SSRI-Resistant Depression in Adolescents; TMS: transcranial magnetic stimulation; UM: ultra-rapid metabolizers; vlPFC: ventrolateral pre-frontal cortex; VNTR: variable number tandem repeat regions; XBP1: X-box binding protein 1; ZNF804A: zinc finger 804A.

## Competing interests

The authors declare that they have no competing interests.

## Authors’ contributions

UO wrote the first draft of the manuscript; UO, CW, and CN made substantial contributions to conception and design of the review, reviewed and revised the first and subsequent drafts for intellectual content. All authors have read and approved the final manuscript.

## Pre-publication history

The pre-publication history for this paper can be accessed here:

http://www.biomedcentral.com/1741-7015/11/132/prepub
